# Holistic Approach to Process Design and Scale-Up for Itaconic Acid Production from Crude Substrates

**DOI:** 10.3390/bioengineering10060723

**Published:** 2023-06-14

**Authors:** Katharina Maria Saur, Robert Kiefel, Paul-Joachim Niehoff, Jordy Hofstede, Philipp Ernst, Johannes Brockkötter, Jochem Gätgens, Jörn Viell, Stephan Noack, Nick Wierckx, Jochen Büchs, Andreas Jupke

**Affiliations:** 1 Fluid Process Engineering (AVT.FVT), RWTH Aachen University, 52074 Aachen, Germany; 2 Biochemical Engineering (AVT.BioVT), RWTH Aachen University, 52074 Aachen, Germany; 3 Process Systems Engineering (AVT.SVT), RWTH Aachen University, 52074 Aachen, Germany; 4 Forschungszentrum Jülich, Institute of Bio- and Geosciences IBG-1, 52428 Jülich, Germany

**Keywords:** biorefinery, scale-up, itaconic acid, *U. cynodontis*, conceptual process design

## Abstract

Bio-based bulk chemicals such as carboxylic acids continue to struggle to compete with their fossil counterparts on an economic basis. One possibility to improve the economic feasibility is the use of crude substrates in biorefineries. However, impurities in these substrates pose challenges in fermentation and purification, requiring interdisciplinary research. This work demonstrates a holistic approach to biorefinery process development, using itaconic acid production on thick juice based on sugar beets with *Ustilago* sp. as an example. A conceptual process design with data from artificially prepared solutions and literature data from fermentation on glucose guides the simultaneous development of the upstream and downstream processes up to a 100 L scale. Techno-economic analysis reveals substrate consumption as the main constituent of production costs and therefore, the product yield is the driver of process economics. Aligning pH-adjusting agents in the fermentation and the downstream process is a central lever for product recovery. Experiments show that fermentation can be transferred from glucose to thick juice by changing the feeding profile. In downstream processing, an additional decolorization step is necessary to remove impurities accompanying the crude substrate. Moreover, we observe an increased use of pH-adjusting agents compared to process simulations.

## 1. Introduction

Bio-based bulk chemicals such as carboxylic acids, amines and alcohols play an important role in the transition towards a circular bioeconomy [1]. They can potentially replace fossil-based chemicals in the packaging, food and pharmaceutical industry or build new functional materials [2,3]. However, the market share of bio-based bulk chemicals in 2019 was only at 1–2%, as they struggle to compete with fully integrated and established petrochemical processes [4]. Substrate availability is one primary impediment to implementing economically feasible production processes. While pure substrates are easy to process, they are predominantly destined for the food and feed industry and typically only used for small-scale, high-value biotechnological processes [4]. Using crude substrates from the food and agricultural industry provides a promising alternative [2,4,5,6,7,8].

For some processes, such as bio-ethanol production or baker yeast fermentation, using crude substrates is state-of-the-art [9,10]. In these processes, the product is usually captured by unit operations such as filtration and overhead distillation, where impurities originating from feedstocks play a minor role. However, in the field of organic acid production, the use of complex feedstocks poses additional challenges, as potential impurities such as other organic acids, pigments, salts and preservatives [11] can influence downstream processing (DSP) [12,13,14]. Furthermore, these impurities can also impact fermentation performance [15,16]. Thus, fermentation protocols tailored to crude substrates [6], reliable online analytics and efficient separation methods [17] must be developed and aligned to create sustainable and economically feasible processes [4].

This work presents an interdisciplinary method for process development, using itaconic acid (ITA) production with *Ustilago* sp. as a primary example. In contrast to an independent development of single unit operations, fermentation, online analytics and DSP are established in parallel and are guided by a conceptual process design. This allows a close collaboration of these three fields and a rapid process implementation. At first, a purification route is chosen that is suited for selective ITA separation from a broth with a multitude of side components. The process is modeled using Aspen Plus (V11) (Aspen Technology, Inc., Bedford). The model uses key performance indicators (KPI) from fermentations on glucose for different *Ustilago* sp. [18,19], to identify a cost-efficient organism [20]. Subsequently, the process with the favored organism is experimentally investigated at laboratory-scale, using real substrates. Ultimately, the process is scaled to 100 L fermentation volume and compared with laboratory-scale results. Challenges in bioprocess design with crude substrates are discussed and further optimization potential is described.

As a dicarboxylic acid with a methylene group, ITA is primarily used as a cross-linker in synthetic latex production [21,22]. However, if the production costs are reduced, it may be used as a versatile and promising starter molecule in the production of polymers and hydrogels [23,24,25,26,27]. Considerable potential also lies in replacing malic acid anhydride in polyester resin production [28,29,30]. This broad application potential makes ITA a suitable exemplary target compound for this work. Furthermore, it offers the opportunity to evaluate the influence of new production hosts and crude substrates on DSP feasibility.

Since the 1950s, ITA has been produced with *Aspergillus terreus* by batch fermentation [31,32]. Fermentations found in the literature reach industrially relevant titers and productivities of up to 160 g/L [33] and 1.15 g/(L·h), respectively, [34]. Alternatively, the process can be designed for high substrate yields of up to 0.715 g_ITA_/g_glucose_ [35]. Nevertheless, ITA production with *A. terreus* remains challenging, as extensive morphological control and, in the case of crude substrates, pretreatment is necessary for high productivity [31,33,36,37]. Additionally, *A. terreus* is classified into biosafety level 2 in some countries [38], making the implementation of biorefineries difficult. Recent efforts to circumvent these issues target the development of new production hosts [39,40,41]. Especially natural producers such as *Ustilago* sp. with their broad substrate spectrum and yeast-like growth profile show great potential [19,42,43,44,45,46,47,48,49]. In this work, two ITA producing organisms are compared in a techno-economic analysis demonstrating the effect of fermentation pH, yield and product titer on process feasibility. For fermentation at neutral pH, *Ustilago maydis* MB215 Δcyp3 ΔMEL ΔUA Δdgat ΔP_*ria*_::*P_etef_* Δfuz7 *P_etef_ mttA*_K14 (*U. maydis* K14) from Becker et al. (2020) [19] is used. *Ustilago cynodontis* NBRC 9727 Δfuz7 Δcyp3 *P_etef_mttA P_ria1_ria1* (*U. cynodontis* ITA Max pH) has the benefit of being much more acid-tolerant than *U. maydis* [18] and is therefore used to evaluate fermentation at low pH values. The most promising production organism from these strains is selected for experiments on real substrates.

In contrast to defined carbon sources, crude substrates differ enormously in their composition and seasonal availability [11,50]. They also vary regarding their regional availability [4]. As a result, transportation costs can be a deciding factor for the economic feasibility of biorefineries [51] and a regional implementation shows excellent potential. This work focuses on substrates available for a decentralized biorefinery in the German Rhineland. The region has a large agricultural sector and faces structural and economic changes due to phasing out of lignite mining. Before starting this research, multiple crude substrates are considered regarding their suitability and impurities. For this study, thick juice produced by sugar beet processing is selected as feedstock. Utilizing thick juice to produce ITA is favorable opposed to additional purification for the isolation of lower-value sucrose. Furthermore, it is locally available in large quantities and has a high sugar concentration. It also shows only minor impurities such as lactic acid and pigments, which nevertheless reflect typical challenges when processing crude substrates (Appendix A).

## 2. Materials and Methods

### 2.1. Fermentation and Cultivation

For fermentation and cultivation, all chemicals are purchased from Carl Roth GmbH & Co. KG (Karlsruhe, Germany). *U. cynodontis* NBRC 9727 Δfuz7 Δcyp3 *P_etef_mttA P_ria1_ria1* is used for all cultivation experiments [44]. The composition of thick juice used for all cultivations can be found in Appendix A.

#### 2.1.1. Cultivation Media

Cultivations are performed in a modified Verduyn medium as described by Geiser et al. (2014) [43]. The medium is composed of 0.8 g/L NH${}_{4}$Cl, 0.2 g/L MgSO${}_{4}\cdot$7 H${}_{2}$O, 0.01 g/L FeSO${}_{4}\cdot$7 H${}_{2}$O, 0.5 g/L KH${}_{2}$PO${}_{4}$, 0.1 vol% trace element solution and 0.1 vol% vitamin solution. The trace element solution contains 15 g/L TitriplexIII ©, 4.5 g/L ZnSO${}_{4}\cdot$7 H${}_{2}$O, 0.84 g/L MnCl${}_{2}\cdot$2 H${}_{2}$O, 0.3 g/L CoCl${}_{2}\cdot$6 H${}_{2}$O, 0.3 g/L CuSO${}_{4}\cdot$5 H${}_{2}$O, 0.4 g/L Na${}_{2}$MoO${}_{4}\cdot$2 H${}_{2}$O, 4.5 g/L CaCl${}_{2}\cdot$2 H${}_{2}$O, 3 g/L FeSO${}_{4}\cdot 7$ H${}_{2}$O, 1 g/L H${}_{3}$BO${}_{3}$ and 0.1 g/L KI. The vitamin solution contains 0.05 g/L D-biotin, 1 g/L D-calcium pantothenate, 1 g/L nicotic acid, 25 g/L *myo*-inositol, 1 g/L thiamine hydrochloride, 1 g/L pyridoxine hydrochloride and 0.2 g/L para-aminobenzoic acid. Flask pre-cultures contain 0.03 M 2-(N-morpholino)ethanesulfonic acid as a buffer at pH 6.5. Stirred tank reactor cultivations are supplemented by 1 g/L yeast extract and 0.05 vol% Antifoam 204. All media components are either autoclaved or sterile-filtered with a 0.2 µm cut-off filter (Millipore-Sigma, Burlington, VT, USA). Thick juice from sugar beets is kindly provided by Pfeifer & Langen GmbH & Co. KG (Jülich, Germany). It is autoclaved in undiluted form and used as the sole carbon source.

#### 2.1.2. Cultivation Conditions

*U. cyndontis* ITA Max pH is stored in cryogenic cultures at -80 °C, containing 30 vol% of 500 g/L glycerol stock and 70 vol% culture grown on modified Verduyn medium. For each fermentation, one YEPS plate with 10 g/L yeast extract, 10 g/L peptone, 10 g/L sucrose and 20 g/L agar-agar is inoculated from one fresh cryogenic vial and cultivated at 30 °C for 48 h. From this plate, 500 mL shake flasks with 50 mL filling volume, containing Verduyn medium with 50 g/L sucrose from thick juice and 2 g/L NH${}_{4}$Cl for growth, are inoculated as pre-culture. The flasks are cultivated overnight at 30 °C and 300 rpm with a shaking diameter of 50 mm. The filling volume is then centrifuged in a Rotina 35 R centrifuge (Andreas Hettich GmbH & Co. KG, Tuttlingen, Germany) at 4000 rpm for 10 min. The pellets are suspended in 3 mL of the resulting supernatant and the fermentation is inoculated to an optical density (OD${}_{600}$) of 0.25.

Small-scale cultivations are performed in a 2 L Sartorius BIOSTAT^®^ stirred tank reactor (Sartorius AG, Goettingen, Germany) equipped with a six-blade Rushton turbine with a diameter of 45 mm and four baffles. The initial filling volume is 1 L and the temperature is kept at 30 °C. Dissolved oxygen tension (DOT) is regulated at >30% by increasing the stirring frequency from 400 or 800 to 1200 rpm. pH is controlled by adding 5 M NaOH or 2.5 M Mg(OH)${}_{2}$. Due to its low solubility in water, 2.5 M Mg(OH)${}_{2}$ is pumped in a cycle to avoid settling of the crystals within the tubing for the respective fermentation. A second pump delivers the Mg(OH)_2_ solution from the primary cycle to the fermenter. Off-gas composition is determined with a DASGIP off-gas analyzer GA4 (Eppendorf SE, Hamburg, Germany). pH and DOT are measured using an Easyferm plus PHI K8 200 (Hamilton, Hoechst, Germany) and a VisiFerm DO ECS 225 probe (Hamilton, Hoechst, Germany), respectively.

Pre-cultures for the 100 L fermentation scale are performed in 2 L Sartorius fermenters as described above. The pre-culture is run with 50 g/L sucrose from thick juice and 2 g/L NH${}_{4}$Cl. After 24 h, the whole filling volume is used to inoculate a 100 L fermenter (Frings GmbH, Rheinbach, Germany) which is equipped with three six-blade Rushton turbines with a diameter of 150 mm and four baffles. Cultivations are performed with an initial filling volume of 105 L at 30 °C and a stirring rate of 285 rpm. pH is controlled by adding 5 M NaOH. OTR and CTR are determined by measuring the off-gas with a Rosemount™X-STREAM XEFD exhaust gas analyzer (Emerson Automation Solutions, Langenfeld, Germany). pH and DOT are monitored using a Polylite Plus H VP 120 Pt100 (Hamilton, Hoechst, Germany) and a VisiPro DO Ex 120 H2 probe (Hamilton, Hoechst, Germany).

Yield is calculated in g_ITA_/g_sucrose_ and also converted to the amount of glucose equivalents in g_ITA_/g_glucose eq._. For the calculation of glucose equivalents, it is assumed that water hydrolyzes sucrose into glucose and fructose and the utiliziation of fructose proceeds similarly to glucose. Both values are calculated using mass balances. Space-time yield (STY) is also calculated using mass balances. As a critical fermentation parameter, the respiratory quotient (RQ) [-] is calculated dividing the CO_2_ transfer rate (CTR) by the O_2_ transfer rate (OTR). These parameters are used to draw conclusions on the organism’s metabolic behavior, as has previously been demonstrated [52,53,54] (Appendix B). Samples taken during the cultivations are included in the mass balance calculations.

#### 2.1.3. Offline Analytics

Samples for offline analysis are drawn from fermentation at regular intervals. All analyses are done in triplicates. For each sample, 2 mL fermentation broth is centrifuged at 14,000 rpm for 10 min in a Sigma 1–15 centrifuge (Sigma Laborzentrifugen GmbH, Osterode am Harz, Germany). The supernatant is filtered with a 0.2 µm cut-off filter (Millipore-Sigma, Burlington, MA, USA) and analyzed for sugars and organic acid content by high performance liquid chromatography (HPLC). HPLC analysis is performed using a Thermo Fisher Ultimate 3000 (Thermo Fisher Scientific Inc., Waltham, MA, USA) equipped with an ERC RefractoMax 520 RID (Shodex, Munich, Germany). For separation, a ROA-Organic Acid H+ (8%) column (300 × 7.8 mm) (Phenomenex, Torrance, USA) column is heated to 30 °C and used with a mobile phase of 5 mM H${}_{2}$SO${}_{4}$ running at 0.8 mL/min. Cell pellets are dried at 80 ° C for 48 h and then weighed to determine cell dry weight (CDW).

### 2.2. Downstream Processing

Chemicals are purchased from VWR International GmbH (Radnor, PA, USA). Decolorization resins are obtained from Fisher Scientific GmbH (Schwerte, Germany), carbon powder activated is purchased from Thermo Fisher GmbH (Kandel, Deutschland) and activated vegetal charcoal from VWR International GmbH (Randnor, PA, USA). If not stated otherwise, all experiments are performed in triplicates.

#### 2.2.1. Determination of Solubility for ITA and ITA Salts

For solubility measurements, 1 mol ITA is added to an aqueous solution of 1 L (deionized water or fermentation broth) in a 10 mL scale. To ensure full protonation of ITA, 200 µL 25 wt% M H_2_SO_4_ is added. The samples are stirred for 2.5 h in a water bath at defined temperatures. Subsequently, the supernatant is collected by filtration with a CHROMAFIL^®^ Xtra H-PTFE 20/25 0.2 µm filter (MARCHERY-NAGEL GmbH & Co. KG, Düren, Germany). The amount of dissolved ITA is analyzed by HPLC. Solubility of MgITA, Na_2_ITA, K_2_ITA and CaITA is measured at pH > 7.0 to ensure only precipitation of ITA salts. Measurements are performed at 20 °C. For each salt, ITA and the base of the corresponding cation (Mg(OH)_2_, NaOH and Ca(OH)_2_) are combined step-wise according to the stoichiometry of the precipitating salt. The supernatant is analyzed by HPLC (Section 2.2.4) after each addition of ITA. The maximum solubility of the salt is reached when the ITA concentration in the supernatant does not increase further.

#### 2.2.2. Development of a Decolorization Protocol

To screen decolorization agents, resins and charcoals are washed with 20 mL distilled water per g resin. Subsequently, 95% of the washing water is removed and an artificial screening solution is added. For the artificial screening solution, 260 g/L thick juice and 50 g/L ITA are combined and the pH is adjusted to 2.0 with H_2_SO_4_, corresponding to the pH after the first cooling crystallization step and the cooling crystallization of the associated mother liquor. The thick juice concentration corresponds to the production of 50 g/L ITA at a yield of 0.3 g_ITA_/g_glucose eq._. This composition leads to a high amount of impurities in relation to ITA, thus enabling the identification of efficient decolorization agents. The samples are incubated for at least 4 h on a LS-W orbital shaker (Küner AG, Birsfelden, Switzerland) at room temperature. Subsequently, the samples are centrifuged and vacuum filtrated at 0.2 bar with a CHROMAFIL^®^ Xtra H-PTFE 20/25 0.2 µm filter (MARCHERY-NAGEL GmbH & Co.KG, Düren, Germany). To determine the degree of decolorization, absorption between 450 nm and 700 nm is measured and referenced to the solution before incubation with decolorization agents. To quantify ITA adsorption, samples are analyzed by HPLC (Section 2.2.4) before and after incubation (Appendix C).

#### 2.2.3. Processing of Real Fermentation Broth

For laboratory-scale purification, the fermentation broth is centrifuged with a Rotina 35 R centrifuge (Andreas Hettich GmbH & Co. KG, Tuttlingen, Germany) at 4000 rpm for 10 min and then filtrated through a Merck Steritop^TM^ 0.2 µm filter (Merck KGaA, Darmstadt, Germany). Concentration is performed by rotary evaporation with an IKA^®^ RV10 auto HB rotary evaporator and an IKA^®^ VACSTAR digital vacuum pump (IKA^®^-Werke GmbH & Co. KG, Staufen, Germany) at 60–65 °C and 100 mbar. For the main crystallization steps, ITA is concentrated to 316–395 g/L. For crystallization of the mother liquor, ITA is concentrated to 103–106 g/L. Subsequently, crystallizations are conducted using an EasyMax 102 Titration Calorimeter (Mettler Toledo, Columbus, OH, USA). A cooling rate of 0.3 K to reach 15 °C starting from 68 °C is applied. The stirrer speed is 300 rpm. The pH is controlled at pH 2.8 by adding 5 M HCl with a SIMDOS^®^ O2 FEM 1.02 S pump (KNF Holding AG, Sursee, Switzerland) controlled by an InLab^®^ Semi Micro pH electrode (Mettler Toledo, Columbus, OH, USA). The resulting crystals are separated from the mother liquor by filtration with a Whatman Grade 50 Thin filter (Cytiva Europe GmbH, Freiburg im Breisgau, Germany) and dried with a VT 6060 M VACUTHERM vacuum oven (Fisher Scientific GmbH, Schwerte, Germany) at 40 °C and 200 mbar vacuum for 72 h. For decolorization of the crystals, crystals are pooled and dissolved to a concentration of 80 g/L. A decolorization agent is added prior to stirring the solution for 4 h with a magnetic stirrer. The decolorization agent is removed by filtration with a Merck Steritop^TM^ 0.2 µm filter (Merck KGaA, Darmstadt, Germany).

For scale-up, the broth from the fermentation in 100 L scale is filtered with a 37 channel atech α-Al${}_{2}$O${}_{3}$ ceramic membrane (Atech Innovations GmbH, Gladbeck, Germany; type 37/3.8, 37 channels, length 1200 mm, outer diameter 41 mm, channel diameter 3.8 mm, pore size 0.2 µL). Filtration is performed with a feed flow of 80 L/min and a transmembrane pressure of 1.5 bar. The membrane is back-flushed with nitrogen for 1 s every minute to prevent cake formation. Evaporation and crystallization are performed using a crystallization system from Normag GmbH (Illmenau, Germany) with a 10 L vessel for crystallization. For pH shift, 30 wt% HCl is used. If not stated otherwise, cooling rates and temperatures are similar to laboratory-scale experiments. After crystallization, crystals are filtered with a 4–7 µm MN 1672 cellulose filter (MARCHERY-NAGEL GmbH & Co. KG, Düren, Germany). Subsequently, crystals are washed using saturated ITA solutions at 15 °C. Decolorization is performed by adding activated charcoal and stirring for 4 h. Finally, the charcoal is removed by filtration with Merck Steritop^TM^ 0.2 µm filters (Merck KGaA, Darmstadt, Germany).

Mass flows are tracked along the process. To close mass balances, samples are analyzed by HPLC, pH and density measurements. All solid fractions are dried after filtration to determine the solid content. The purity of resulting crystals is derived by dissolving crystals in distilled water at a defined volume, followed by HPLC analysis. Yield and purity calculations can be found in Appendix D.

#### 2.2.4. Offline Analytics in Downstream Processing

HPLC analysis is conducted in triplicates with an Agilent 1260 Infinity II (Agilent Scientific Instruments, Santa Clara, CA, USA) setup, using a G7112B binary pump, G7167A multisampler and a G7116A column compartment. For separation, a 5 µL sample is injected and analyzed with a carboxylic acid resin of 10 cm in length and 8 mm in diameter (CS Chromatography Service GmbH, Langerwehe, Germany) and 2.5 mM H_2_SO_4_ as eluent at a flow rate of 1 mL/min at 30 °C. A refractive index detector G7162A (Agilent Scientific Instruments, Santa Clara, CA, USA) is used for detection. Evaluation is performed with the Open Lab Software 3.4.5 (Agilent Scientific Instruments, Santa Clara, CA, USA). pH is measured at room temperature with a SevenCompact pH S220-Basic pH-meter (Mettler Toledo, Columbus, OH, USA) and an InLab^®^ Micro pH electrode (Mettler Toledo, Columbus, OH, USA). Density measurements are conducted with a DMA48 density meter (Anton Paar, Graz, Austria) at 25 °C. Absorption is measured with a Lambda 25 UV/VIS Spectrometer at wavelengths between 450 nm and 700 nm (Perkin Elmer Inc., Waldham, MA, USA) and evaluated with the UV WinLab Software (Version 6.5).

### 2.3. Online Analytics

Raman spectroscopy is performed with a RXN2 Raman analyzer with a 400 mW, 785 nm laser (Endress+ Hauser, Reinach, Switzerland). The system has a fiber-optic cable and 0 mm focal length, 42 cm long and 12 mm diameter immersion optic. Raman spectra for calibration of the quantification models are acquired with 5 s acquisition time and three repetitions using HoloGRAMS version 3.2. Raman spectra pretreatment and IHM are done in PEAXACT version 5.7 (S-PACT GmbH, Aachen, Germany). A linear baseline model is applied. Recorded Raman spectra are evaluated quantitatively using Indirect Hard Modelling (IHM) [55,56,57]. For online measurements in the 100 L fermenter, the probe is fitted with a custom stainless steel adapter to adjust the immersion depth at the bottom of the fermenter vessel. Data are acquired with 1 s acquisition time and 15 repetitions, providing similar intensities to the calibration data but avoiding saturation of the CCD detector of the Raman analyzer. The spectral range is reduced to 800–1800 cm^−1^ to exclude the DOT signal between 1545 and 1565 cm^−1^ that originates from air.

## 3. Results and Discussion

The process development for ITA production on thick juice using *Ustilago* sp. is pursued as follows: Conceptual process design based on parameters derived from the literature and data from artificially prepared solutions, transfer to crude substrates and scale-up. The suggested procedure shall serve as an example for the development of other carboxylic acid processes on crude substrates and pinpoint vital challenges in the design of biorefinery processes.

### 3.1. Conceptual Process Design

At first, a purification route suitable for the selective separation of ITA from a significant fraction of impurities is chosen. An emphasis is put on the selection of the co-salt accumulating along the process due to the use of acid and base for pH control. Subsequently, the process is set up in Aspen Plus (V11) (Aspen Technology, Inc., Bedford, MA, USA). The model is used to pursue an operative cost analysis and to identify the economically most attractive microorganism, thereby guiding further experimental investigations with real substrates. This approach also allows for an evaluation of the main operative cost drivers at an early stage.

#### 3.1.1. Selection of Purification Route

Various purification steps for carboxylic acids can be found in the literature: evaporation [58], extraction [59,60], adsorption/chromatography [61,62], nanofiltration [63], reverse osmosis [63,64], electrodialysis [65], precipitation and crystallization [60,66,67,68]. All of these methods are either concerned with the direct isolation of the carboxylic acid, the separation of the counter-ion accumulated through base addition during fermentation or the removal of water to concentrate the product solution. Independent of the chosen purification strategy, there are common issues to be considered between the different separation processes, such as the fermentation pH, the solubility of the target compound and its salts and the co-salt removal [58]. The evaluation of various process alternatives for the purification of ITA, as in Magalhaes et al. (2017) [69], is beyond the scope of this paper. Therefore, we investigate the challenges resulting from the use of crude substrates in the industrially applied multiple crystallization process as described by Okabe et al. (2009) [22,31]. Additionally, modifications necessary to transfer the process concept for ITA DSP from *A. terreus* to new production hosts are outlined in the following.

**Figure 1:**
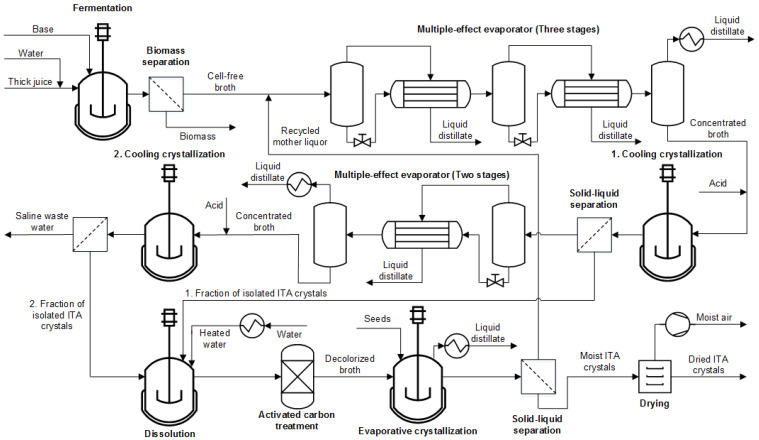
Flowsheet of multiple crystallization process for ITA purification.

The pH during the carboxylic acid production phase in the fermentation is decisive for the ITA DSP. Since *A. terreus* produces ITA at a pH of <3.0 [34], the literature on ITA DSP [22,70] does not explicitly state pH control measures because the amount of base and acid added during fermentation and DSP is negligible. However, ITA fermentation with *U. maydis* and *U. cynodontis* is performed at pH 6.5 and 3.6, respectively, [18,19,71]. Consequently, more base is used to control the pH in fermentation. Additionally, the pH needs to be shifted by inorganic acid addition before the crystallization to protonate ITA species sufficiently. Due to this addition of inorganic base and acid, particular attention needs to be paid to the selection of the co-salt accumulating along the fermentation and purification sequence (Section 3.1.2). Based on these considerations and experimental data obtained from artificially prepared systems (Appendix E), a process flowsheet is derived (Figure 1).

After fermentation, the biomass is separated from the broth via sterile filtration. Subsequently, the broth is concentrated by evaporation. During this step, water is removed from the system so that an ITA concentration of 350 g/L is reached immediately after evaporation. The pH is then adjusted with an inorganic acid. In the crystallization, ITA concentration in the aqueous phase decreases, leading to an increase in pH. Therefore, the amount of pH-adjusting agent is chosen to reach a pH of 2.8 after crystallization. Below a pH of 2.8, ITA solubility stays constant [13] and a yield loss connected to a lack of protonated ITA is avoided. The broth is diluted by adding acid, resulting in an ITA concentration slightly below 350 g/L before crystallization. In the first cooling crystallization step, the temperature is decreased to 15 °C at a rate of 0.3 K/min. The suspension can then be fed to a solid–liquid separator. This sequence is repeated for the mother liquor to increase the overall process yield. However, it must be assured that the broth is concentrated just enough to avoid a simultaneous crystallization of ITA and the co-salt (Section 3.1.2).

Nevertheless, a subsequent purification sequence is necessary to obtain ITA crystals with high purity. Thus, the solid fractions are dissolved again but at an elevated temperature of 80 °C to allow for high ITA concentration and avoid a large heat requirement for water removal in the following process steps. To purify ITA further, the aqueous system undergoes a decolorization treatment. During this step, the temperature is kept at 80 °C preventing simultaneous precipitation of ITA. In the final evaporative crystallization step, water is removed to generate supersaturation. Before initiating evaporation, ITA seeds are added to the solution to allow for a narrow particle size distribution. After crystallization, the mother liquor is recycled to the beginning of the DSP sequence. Residual moisture in the solid fraction is removed by drying.

#### 3.1.2. Identification of Suitable Co-Salt for Multiple Crystallization Process

High-yield crystallization processes require feeds with sufficient product concentration. Thus, the fermentation broth is concentrated along the purification process by evaporation. However, this causes not just a growing concentration of ITA but also of nutrients and a co-salt, which originates from the cation of the base added to the fermenter and the anion of the acid for ITA protonation in DSP (e.g., Na_2_SO_4_). While remaining nutrients are present in low concentrations, the co-salt in carboxylic acid fermentations is present in up to equimolar amounts to the product. To avoid a simultaneous crystallization of ITA and the co-salt, the solubilities of those two components need to differ strongly. Either the co-salt crystallizes first while the product remains in the mother liquor or vice versa. Considering the moderate solubility of ITA [13], a proper co-salt either shows a very low or a very high solubility. The co-salt chosen further specifies the acid and base to be used in the process. Table 1 shows the solubility of common co-salts, which serve as a pool for the following selection process. To allow for a valid comparison between the co-salts, the molar concentration of ITA that can be processed before reaching the solubility limit of the co-salt is calculated and listed as molar ITA-eq. solubility in Table 1. For this, it is assumed that just as much base and acid have to be added to deprotonate and protonate ITA fully. For example, although Na_2_SO_4_ and KH_2_PO_4_ exhibit a similar molar solubility, the amount of ITA processed is more than twice as high for Na_2_SO_4_ than for KH_2_PO_4_ at the solubility limit, which is caused by phosphoric acid delivering only one proton to the system in the relevant pH range, while sulfuric acid provides two. Before choosing a co-salt from Table 1, the list is narrowed down by considering further criteria described below.

First, all ammonium salts can be excluded, as the product formation phase in ITA fermentation is initiated by nitrogen limitation [18,71]. Second, phosphates and nitrates are significantly more expensive in a direct cost comparison of the common inorganic acids (Table 2). Thus, their corresponding co-salts are also excluded.

Third, any cation fed to the system by using base should not be able to precipitate as ITA salt in the fermenter. While this approach removes ITA from the broth and reduces product toxicity, the system becomes highly viscous and biomass and product salt are hard to separate from each other [19,48]. The solubilities of typical ITA salts are experimentally determined (Table 3). While MgITA, Na_2_ITA, and K_2_ITA are highly soluble, only minor fractions of CaITA suffice to saturate the broth. Due to the inevitable challenges encountered during post-processing, Ca-salts are also excluded from further consideration as co-salt.

With respect to these criteria, no co-salt with very low solubility can be identified from Table 1, which limits the choice of co-salts to highly soluble alternatives and is consistent with the process concept suggested by Okabe et al. (2009) [22]. Within this process, the ITA yield in DSP is limited by the solubility of the co-salt. This advocates a high solubility difference between co-salt and ITA to be most suitable for the process. Consequently, MgCl_2_ can be identified as the most attractive option from Table 1 which sets Mg(OH)_2_ as base to be used in the fermentation and HCl as pH shift agent in the DSP. Additionally, MgCl_2_ offers the possibility to be thermally decomposed, recovering HCl and Mg(OH)_2_, instead of being disposed as saline waste [73].

#### 3.1.3. Selection of Most Promising Production Organism

After a suitable purification route is selected and base/acid species for pH control can be derived from the co-salt selection, the most promising organism among the examined *Ustilago* sp. is determined before real substrate investigations are pursued. In this work, *U. cynodontis* ITA pH Max and *U. maydis* K14 are compared. In contrast to the corresponding wildtype strains, these organisms show no hydroxyparaconate formation, yeast-like growth and a greatly increased ITA production [18,71,74]. Moreover, for *U. maydis* K14, the theoretical yield of 0.72 g_ITA_/g_glucose_ is reached during the non-growing production phase due to a decreased synthesis of (glyco-)lipids [19]. Literature KPIs on the microbial conversion of glucose are provided in Table 4. Those KPIs comprise the pH of fermentation during product formation, final ITA product titer and overall fermentation yield. For both strains, better KPIs are available [18,19], however the KPIs in Table 4 are chosen because they are achieved under very similar pulsed-fed-batch conditions, thus allowing a better comparison.

**Figure 2:**
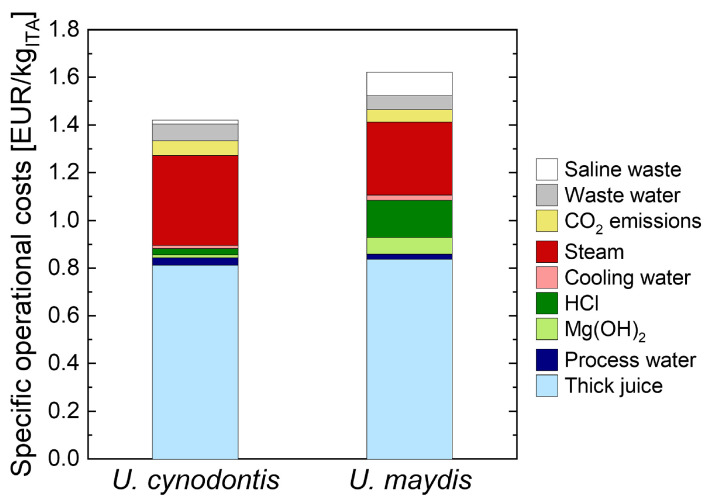
Operative cost analysis of ITA production by *Ustilago* sp.

Based on a direct KPI comparison, no clear decision for one organism can be made, as *U. cynodontis* ITA Max pH can produce ITA at favorable fermentation pH for DSP, but *U. maydis* K14 shows a higher yield and titer. Thus, it is necessary to identify the impact of each KPI on process economics and select the most suitable organism on the grounds of an operative cost analysis. Costs are calculated using the developed flowsheet model in Aspen Plus (Appendix F). Figure 2 displays the results of this analysis.

It becomes apparent that the pH-neutral fermentation of *U. maydis* K14 causes higher costs associated with acid/base use and saline waste. The lower energy requirement due to a higher product titer of *U. maydis* K14 does not compensate those additional costs. Moreover, both organisms’ substrate costs are comparable despite a higher fermentation yield of *U. maydis* K14. This can be explained by a higher DSP yield of 98.1% for *U. cynodontis* ITA Max pH, whereas *U. maydis* K14 attains a purification yield of only 89.8%. The yield difference originates in the second cooling crystallization. To avoid a co-crystallization of MgCl_2_, the preceding evaporation of water is limited. Since *U. maydis* K14 accumulates a larger fraction of co-salt in DSP, the ITA losses in the second cooling crystallization are significantly higher compared to *U. cynodontis* ITA Max pH. Overall, *U. cynodontis* ITA Max pH and *U. maydis* K14 achieve specific operational costs of 1.42 EUR/kg_ITA_ and 1.62 EUR/kg_ITA_, respectively. Therefore, *U. cynodontis* ITA Max pH is considered for further process development with real substrates.

### 3.2. Transfer to Real Systems

The conceptual process design from Section 3.1 is based on literature data obtained from fermentation on glucose [18] and on experiments with artificially prepared ITA solutions (Appendix E). In the next step, the process is transferred to real substrates in laboratory-scale to prepare for scale-up and pinpoint technical challenges accompanying biorefinery process development with crude substrates.

#### 3.2.1. Fermentation with *U. cynodontis* ITA Max pH at Laboratory-Scale

**Figure 3:**
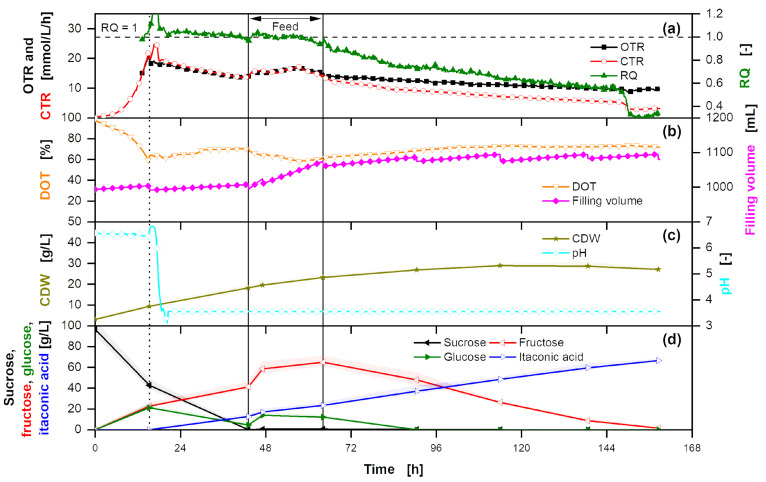
Extended-batch fermentation of *U. cynodontis* ITA Max pH with pH shift and thick juice as carbon source. (**a**) OTR, CTR and RQ. The horizontal dashed line shows RQ = 1. RQ values are only shown for OTR > 10 mmol/(L·h). (**b**) DOT and filling volume. (**c**) CDW and pH. (**d**) Sucrose, glucose, fructose and ITA concentration. For the batch phase, 100 g/L sucrose via thick juice are initially added to the medium. During the feed phase (between the vertical solid lines) a total of 62.1 g of additional sucrose are added into the fermentation vessel. Cultivation is performed in a 2 L stirred tank reactor with an initial filling volume of 1 L at 30 °C with a constant aeration and stirring rate of 1 L/min and 800 rpm, respectively. Drops in the filling volume indicate sampling points. pH is kept above 6.5 or 3.6 before and after the pH shift, respectively, by addition of 5 M NaOH. For clarity, only every 15th measured online data point is shown.

Following the techno-economic analysis (Section 3.1.3), a laboratory-scale fermentation is performed with *U. cynodontis* ITA Max pH as production organism (Figure 3) using a low nitrogen concentration [18]. Next to substrate concentration as well as ITA and biomass production, OTR, CTR and RQ are monitored. Biomass formation on glucose results in a RQ slightly above 1, while ITA production has a theoretical RQ of 0.66 (Equations (A7) and (A8)). The use of sucrose as the primary carbon source and the influence of impurities (Appendix A) on fermentation are discussed in the following.

ITA production is initiated by nitrogen limitation, dividing ITA fermentation into a growth and production phase [44,71]. On glucose, the growth phase of *U. cynodontis* ITA Max pH is not strongly affected by pH and can be conducted equally at pH 6.5 and 3.6 [74]. With thick juice as a substrate, even the acid-tolerant *U. cynodontis* ITA Max pH did not grow properly, which might be attributed to the lactic acid present in thick juice (Appendix A) exhibiting weak organic acid stress during the growth phase [75]. Therefore, the pH is kept at >6.5 at the beginning of the fermentation to ensure improved growth. The resulting exponential growth phase is mirrored in the exponential increase of OTR and CTR (Figure 3). In the work of Tehrani et al. (2019) [18], a drop in CO_2_ production correlated with the start of nitrogen limitation. Therefore, it is assumed that once the CTR decreases, nitrogen limitation initiates product formation. For optimal ITA production, a pH shift is introduced after 15.2 h of fermentation [18]. It allows the pH to drop to 3.6, where it is kept throughout the production phase. Even though nitrogen limitation is reached, the biomass increases further from 9 g/L to 18 g/L between 15.2 h and 43.1 h of fermentation, which is possible because the cells reduce their nitrogen content, as shown by Klement et al. (2012) [76]. The RQ is slightly above 1 and slowly decreasing, showing combined cell growth and increasing ITA production. As thick juice includes additional nitrogen (Appendix A), the biomass is twice as high compared to Tehrani et al. (2019) [18].

Thick juice contains sucrose as the primary substrate, which is cleaved into the monomers glucose and fructose. While up to 43.1 h of fermentation, sucrose is hydrolyzed, a nearly equimolar amount of fructose accumulates, while glucose is used up, making glucose a preferred substrate for ITA production. Fructose is used after glucose is depleted. The fermentation on two different carbon sources necessitates implementing a batch or an extended-batch fermentation for efficient substrate conversion. Feeding thick juice at a limited rate reduces STY, as the substrate uptake rate would be restricted and thus lower. Continuous overfeeding for a constant glucose concentration leads to a reduced substrate yield, as not all substrate is converted over the fermentation time due to product inhibition (Appendix G). Consequently, the feed is started after glucose is nearly depleted at 43.1 h of fermentation and terminated after 64.1 h when 62.1 g sucrose is fed into the fermenter. This termination allows a switch from glucose to fructose metabolism and, thus, a complete substrate conversion until the end of the fermentation. The RQ increases during the feeding, mirroring cell growth on additional nitrogen in thick juice (Appendix A). Simultaneously, the biomass increases further to reach a cumulative concentration of 27 g/L at the end of the fermentation. As both CTR and OTR decline until the end of fermentation, it can be assumed that cell viability also decreases due to weak organic acid stress. At the end of the fermentation, a titer of 66.6 g_ITA_/L and a yield of 0.48 g_ITA_/g_sucrose_ are reached. Converted to glucose equivalents, the yield is at 0.46 g_ITA_/g_glucose eq._. Considering the additional biomass formation from nitrogen in thick juice, this is comparable to fermentations on glucose with a constant feeding profile and an ammonium chloride concentration of 4 g/L [18]. Yield and productivity of the process increase to 0.50 g_ITA_/g_sucrose_ when only the actual production time, starting from the pH shift until the end of the fermentation, is taken into account. This highlights the potential for in situ product removal [53,60], where the production phase is extended, leading to higher fermentation yields.

To increase the overall process yield, MgOH_2_ provides a viable alternative as pH-adjusting agent, as the fermentation broth can be further concentrated in the 2nd cooling crystallization (Section 3.1.2). To investigate the tolerance of *U. cynodontis* ITA Max pH towards Mg(OH)_2_ [58], an extended-batch fermentation on thick juice with *U. cynodontis* ITA Max pH is carried out with MgOH_2_ as a base (Appendix H). While growth and ITA production are possible, Mg(OH)_2_ shows low solubility in water at high pH values and is fed as a suspension, which presents challenges, especially regarding the blocking of tubing and pipes. Due to the increased difficulty in handling, further scale-up experiments are conducted with NaOH as a base to alleviate the proof-of-concept.

#### 3.2.2. Decolorization Protocol

After fermentation with glucose, the fermentation broth shows a golden color. Two crystallization steps are sufficient to produce white crystals. On the contrary, fermentation broth with thick juice is of a dark brown shade due to multiple colored substances being present in the crude substrate (Appendix A and Appendix I). Crystals obtained from this more complex fermentation broth show a light to dark brown color as the pigments are incorporated into the crystals. Even though some of these pigments can be removed by applying multiple crystallization steps, as illustrated in Appendix I, developing a decolorization step is essential to obtain white crystals. While in the sugar industry, decolorization is usually implemented using anion exchange resins [77,78], decolorization in organic acid production is most commonly realized by adding activated charcoal [22,79,80,81]. However, similar to other carboxylic acids, ITA can adsorb at both types of these decolorization agents, reducing process yield and thereby economic feasibility [82] (Section 3.1.1). In this work, the potential yield loss is minimized by two measures. First, the decolorization step is located after dissolving ITA crystals obtained from the first cooling crystallization step and the cooling crystallization of the mother solution as described by Okabe et al. (2019) [22] (Section 3.1.1). As some pigments remain in the mother liquor of the crystallization steps, less decolorization agent is necessary. Consequently, less ITA can be removed from the process by adsorption to a decolorization agent. Second, different decolorization agents from both sugar and organic acid production are screened for sufficient pigment removal and minimum product loss. Two strong basic anion exchange resins and one weak basic anion exchange resin with a more hydrophobic binding site are compared to two kinds of activated charcoals. Additionally, one hydrophobic resin is compared to the activated charcoals (Table 5). Details regarding developing a screening protocol and the absorption behavior of thick juice can be found in Appendix C.

To enable parallel development of cultivation and DSP, an artificial broth at pH 2 (Section 2.2.2) is used for screening experiments. In the final process, the solution would be heated to 80 °C to facilitate the dissolution of ITA (Section 3.1.1) and to potentially reduce ITA adsorption (Appendix J). Nevertheless, due to potential difficulties in handling, especially in scale-up, experimental decolorization is conducted at 20 °C.

**Figure 4:**
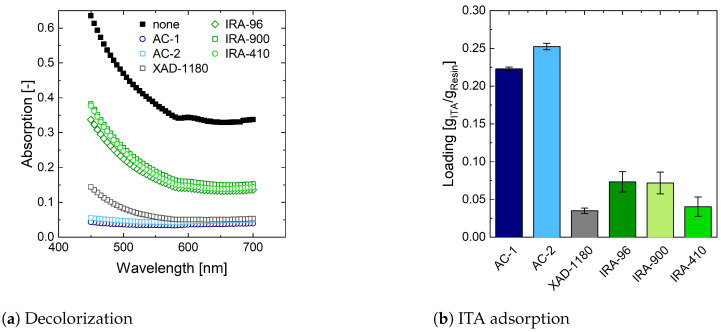
Identification of suitable decolorization agents. (**a**) Decolorization of artificially prepared broth with 50 g/L decolorization agent. (**b**) ITA adsorption from artificially prepared broth. Artificially prepared broth consists of 50 g/L ITA and 260 g/L thick juice at pH 2.0. The screening is performed at 20 °C.

Figure 4a depicts the decolorization behavior of different decolorization agents. Both activated charcoals show satisfactory performance at wavelengths between 500 and 700 nm. At higher wavelengths, absorption showed a slight increase. The XAD-1180 resin depicts a similar behavior (Figure 4a). Anion exchange resins, however, cannot sufficiently decolorize the artificial broth at a resin concentration of 50 g/L. This can be correlated to the pigment composition in thick juice. They are expected to be of a hydrophobic nature at low pH values and thus not adsorb at ionic decolorization agents (Appendix A) [78,83,84]. Figure 4b illustrates ITA adsorption on decolorization agents. Activated charcoals adsorb up to 0.20 ± 0.02 g_ITA_/g_resin_ from artificially prepared broth, whereas the also hydrophobic XAD-1180 only adsorbs 0.04 ± 0.01 g_ITA_/g_resin_. Characterization of ITA adsorption in artificially prepared systems reveals that mostly undissociated ITA is adsorbed on hydrophobic decolorization agents. Furthermore, ITA adsorption in artificially prepared systems is higher compared to the artificially prepared broth, indicating competitive adsorption of ITA and pigments (Appendix K). Corresponding to literature data [85], weak and strong anion exchange resins adsorb ITA. Combining this potential product loss with low decolorization performance, anion exchange resins are not further considered in the process development. XAD-1180, however, shows low ITA adsorption and good decolorization and is thus chosen for further experiments. Even though activated charcoals show an increased ITA adsorption, they also perform very well in decolorization. Therefore, XAD-1180 is measured against the best-performing charcoal AC-1.

**Figure 5:**
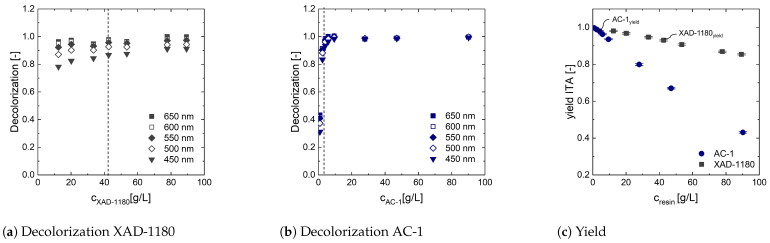
Amount of resin needed for XAD-1180 and AC-1 to sufficiently decolorize crystals after the first and second pH shift cooling crystallization. (**a**) Decolorization with XAD-1180. (**b**) Decolorization with AC-1. Dashed lines indicate the proposed resin concentration for sufficient decolorization. (**c**) ITA yield depending on resin concentration for XAD-1180 and AC-1.

To successfully implement decolorization into the process, the minimum amount of decolorization agent necessary is determined for AC-1 and XAD-1180. The re-dissolved crystals from fermentation on thick juice (Figure 3) are incubated with different resin concentration. Decolorization and yield loss are investigated. Figure 5a shows that even at high concentration, XAD-1180 cannot fully decolorize the ITA solution. Especially compounds absorbing light at low wavelengths remain in the solution as some pigments barely interact with the strictly hydrophobic XAD-1180 resin. Activated charcoals such as AC-1, on the other hand, contain multiple binding sites and can adsorb pigments with a wide variety of physiochemical properties (Figure 5b) [77,78,83]. As a result, only 4 g/L AC-1, but at least 40 g/ XAD-1180 are necessary to obtain a sufficient decolorization. When correlating the amount of resin to the potential yield loss as shown in Figure 5c, it is apparent that this would lead to a yield loss of 2.16 ± 0.02% for AC-1 and 7.00 ± 0.39% for XAD-1180. Accordingly, AC-1 at a concentration of 4 g/L is selected for the decolorization step. These findings highlight activated charcoals as promising candidates for decolorizing fermentation broth with crude substrates from the sugar industry. In future experiments, regeneration of activated charcoals, e.g., by solvents [86] or biological methods [87] must be evaluated.

#### 3.2.3. Downstream Process Evaluation on Real Substrates at Laboratory-Scale

To prepare for scale-up, the fermentation broth produced in Section 3.2.1 is purified once by multiple crystallization in laboratory-scale. Besides using NaOH as a base, other process design specifications outlined in Section 3.1.1 and Section 3.1.2 are selectively amended to facilitate the laboratory-scale experiments. Instead of a continuous processing, the broth is purified sequentially in a batch-wise operation of the individual unit operations. Thus, a rotary evaporator and a titration unit are used for evaporation and crystallization, respectively. The titration unit is able to perform the cooling crystallization and the pH shift simultaneously. Appendix L shows an exemplary cooling crystallization profile. However, evaporative crystallization cannot be performed with the above applied equipment due to the separation of evaporation and crystallization. Therefore, the final evaporative crystallization is replaced by a third cooling crystallization at the present scale. The recycling of the mother liquor after the final crystallization is omitted due to the batch-wise processing of the fermentation broth. As stated in Section 3.2.2, dissolution and decolorization are operated at 20 °C instead of 80 °C.

The first and final crystallization step show similar yields of 91.62% and 93.64%, respectively. While no pH-adjusting agent is needed in the final crystallization, 300.4 mL 5 M HCl per liter of concentrated ITA solution are added to maintain a pH of 2.8 in the first pH-shift cooling crystallization (Appendix L). For the cooling crystallization of the mother liquor from the first crystallization, the yield is at 67.50%. The decrease in yield is caused by the limited evaporation necessary to avoid co-salt precipitation. As stated in Section 3.1.2, this decreases crystallization yield.

To evaluate the influence of crude substrates and fermentation-based side compounds, the expected crystallization yields calculated based on solubility data and the yields obtained at laboratory-scale are compared in Appendix E. If ITA solubility in artificially prepared systems is used for yield calculation, the experimental yields are well above the expected values. However, ITA solubility decreases in fermentation broth with thick juice. This may be due to the influence of salts from the fermentation medium and impurities from the substrate [13]. Furthermore, an increase in the use of pH-adjusting agents due to buffering substances in the fermentation broth can also contribute to changes in solubility (Section 3.3.3). Consequently, if solubility data from complex systems is used for yield calculations, predictions are more accurate. However, this is still insufficient to fully depict ITA yields in crystallization as water is evaporated to increase ITA concentration. This also leads to an increased concentration of side compounds not portrayed in solubility experiments with fermentation broth from crude substrates (Appendix E).

For decolorization, the yield is at 93.65%. As expected, the crystal purity of the dry solid fractions is slightly increasing over the process sequence from 97.65% in the first cooling crystallization to 98.57% in the final cooling crystallization. This is mirrored in crystal coloration (Appendix I). In future studies, recycling the mother liquor has to be considered to evaluate the influence of salts accumulating in the crystallization process on yield and purity.

### 3.3. Scale-Up and Comparative Assessment

The results from the conceptual process design and real substrate investigations in the laboratory are used to perform a scale-up to a fermentation broth volume of 100 L to demonstrate the feasibility of the production process for industrial application.

#### 3.3.1. Scale-Up of Fermentation Process

**Figure 6:**
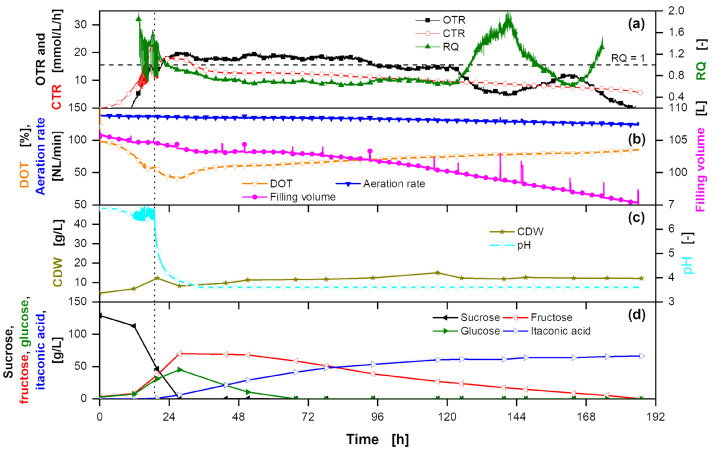
100 L batch fermentation of *U. cynodontis* ITA Max pH with pH shift and thick juice as sole carbon source. (**a**) OTR, CTR and RQ. The horizontal dashed line shows RQ = 1. RQ values are only shown for OTR values > 10 mmol/(L·h). (**b**) DOT, absolute aeration rate and filling volume. (**c**) CDW concentration and pH. (**d**) Sucrose, glucose, fructose and ITA concentration. 137 g/L sucrose from thick juice are initially added to the medium. Cultivation is performed in a 150 L stirred tank pressure reactor with an initial filling volume of 105 L at 30 °C with a stirring rate of 285 rpm and gauge pressure of 300 mbar. pH is controlled by addition of 5 M NaOH.

The fermentation at elevated scale is performed using NaOH as a base. To allow for scale comparability, the stirring frequency is adapted to keep the volumetric power input constant during scale-up [88]. The aeration, however, is set to 1 vvm as in laboratory-scale experiments. As the volume of the fermenter decreases throughout operation due to evaporation, the aeration is adjusted accordingly. The filling volume is determined via the weight of the fermenter and spikes in the filling volume are visible when samples are taken (Figure 6). There is no effect of substrate overfeeding on *U. cynodontis* ITA Max pH metabolism observed in this work. Additionally, sufficient oxygen transfer can be realized (Appendix G). Thus, a batch process with an initial substrate concentration of 137 g_sucrose_/L from thick juice is performed to facilitate the first scale-up. Similar to laboratory-scale experiments, biomass formation, ITA production, OTR, CTR and RQ are monitored to reflect the overall metabolic activity (Figure 6). The following section will discuss the effects of the scale-up and the switch to batch fermentation mode.

The first 20 h of fermentation are comparable to fermentation in laboratory-scale (Section 3.2.1). Fluctuations in CTR are due to changes in pH value, as illustrated in Appendix M. The higher initial CDW is due to inoculation with 1 vol% instead of a specific OD. However, after the initiation of the nitrogen limitation, no increase in biomass is observed, resulting in a low CDW of 12.1 ± 0.4 g/L. The inoculation with a fully grown culture can partially explain the deviation from laboratory-scale experiments. The cells already experienced nitrogen limitation and may have changed their composition to adapt to nitrogen-limited conditions [76]. This difference in metabolic state is also reflected in OTR and CTR. Between 24 h and 125 h the CTR and OTR are decreasing from 17.8 to 9.4 mmol/(L·h) and from 18.2 to 11.4 mmol/(L·h), respectively. This leads to a constant RQ of approximately 0.8, indicating increased product formation compared to small-scale fermentation. As a result, nearly double the amount of ITA per g CDW is produced and the productivity nominated to CDW is higher as in laboratory-scale (Table 6). However, the fermentation attains a yield of 0.46 g_ITA_/g_sucrose_ (corresponding to 0.44 g_ITA_/g_glucose eq._) indicating more substrate being consumed for cellular maintenance, due to weak organic acid stress [89] and byproduct formation [90,91], which occurs to a large extent at the end of the fermentation. After 116.7 h, only 263 g ITA are produced, while 2.6 kg fructose are consumed. A sole substrate conversion for maintenance is unlikely due to the short time frame and no increase in the CTR. However, it has previously been shown that *U. cynodontis* can produce glycolipids [90,91] and, thus, more reduced hydrocarbons may be formed. This hypothesis is supported by the drop in the OTR, leading to an RQ well above 1.

The fermentation is terminated based on Raman spectra obtained during fermentation as described below (Section 3.3.2). At the end of the fermentation, 6.4 kg ITA at a STY of 0.35 g/(L·h) are produced. Considering overall yield, STY and titer, the batch fermentation at 100 L scale and the extended-batch fermentation in laboratory-scale behaved similarly (Table 6). The KPIs show the feasibility of the scale-up of an ITA fermentation with thick juice as the sole carbon source in batch mode.

#### 3.3.2. Possibility of Fermentation Control by Raman Spectroscopy

As other crude substrates possibly show inhibitory effects, extended-batch fermentations should be established in the future. The termination of the feed needs to be closely timed to ensure a complete substrate conversion before product inhibition sets in. As crude substrates are subject to seasonal fluctuations that can influence the conversion rate and process yield [11], online analytics are indispensable for accurate feeding. Optical methods such as Raman spectroscopy show great potential, as they do not interfere with the process and provide highly time-resolved data [92]. However, the Raman spectra of various compounds overlap and more than simple univariate chemometric models are needed to translate the spectra into concentration data [92]. Nevertheless, Raman spectroscopy can be combined with indirect hard modeling (IHM) to quantify compounds with overlapping Raman bands [93]. To demonstrate the general feasibility of this method for biorefinery applications, the IHM approach from Echtermeyer et al. (2021) [93] for ITA is extended with sucrose, fructose and glucose (Appendix O). The coefficient of determination for all components is close to 1, indicating that the mixture hard model covers the underlying variance in the calibration data well (Table A4).

**Figure 7:**
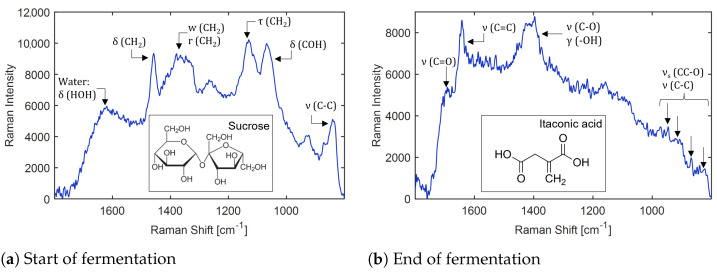
Assignment of molecular vibrations to the Raman spectrum of fermentation broth, recorded during the 100 L fermentation. (**a**) At 0 h, the broth contains 137 g/L sucrose and no product, as confirmed by the presence of sucrose bands. (**b**) At 187 h, broth contains 66.4 g/L ITA and no sugars, as confirmed by the presence of ITA bands. A linear baseline has been applied to both spectra. ν: stretching, ν_s_: symmetric stretching and γ: deformation modes.

To evaluate the potential of controlling fermentation parameters by Raman spectroscopy, the Raman probe is assembled with the 100 L fermenter. Thus, the spectra contain not only the signal of the analytes ITA, glucose, fructose and sucrose but also the signal of other components in thick juice and media. More importantly, fluorescence resulting from thick juice and biomass also contributes to the spectra. As visible in Appendix P, this leads to a broad background that changes its shape over the fermentation time. By applying a linear baseline correction, the influence of fluorescence is reduced, thereby enabling the identification of prominent Raman bands of sucrose, such as the δ_CH_2__ bending vibration at 1458 cm^−1^ or the ν_C-C_ stretching mode at 835 cm^−1^ at the start of the fermentation (Figure 7a). The ν_C=O_ stretching mode at 1697 cm^−1^ for ITA can be seen at the end of the fermentation (Figure 7b). These results enable the termination of the fermentation once sugar is depleted. Nevertheless, the characteristic Raman bands of the analytes are faint. While the model for artificially prepared systems is accurate, it is insufficient for quantitative Raman spectroscopy when dealing with crude substrates such as thick juice in a fermentation process. To establish a feeding profile controlled by Raman makes it necessary to remove the underlying fluorescence, e.g., by using a higher wavelength laser [94] or a mathematical approach, such as polynomial fitting [95] in future research.

#### 3.3.3. Scale-Up of Downstream Process

**Figure 8:**
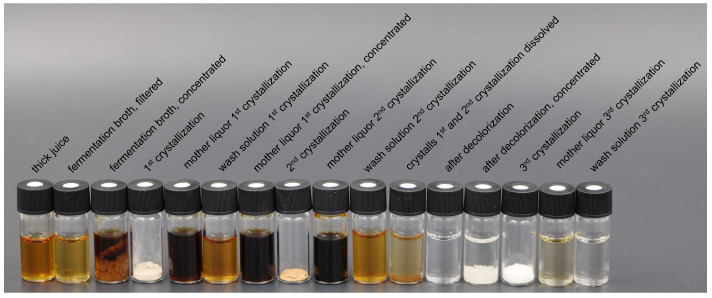
Broth or crystal sample at each process step along the purification sequence.

As in laboratory-scale experiments, the broth is processed in a batch-wise operation without recycling of the mother liquor from the final crystallization. Decolorization is performed at 20 °C (Section 3.2.3). However, evaporation and crystallization are performed in one unit, enabling the implementation of an evaporative crystallization after decolorization. Additionally, ITA-saturated washing solutions remove the remaining solid content from the vessel walls after crystallization. The scale-up is accompanied by process simulations using the Aspen Plus flowsheet presented in Appendix F. The model is applied to determine the expected mass flows at elevated experimental scale and provides a benchmark for measured process variables. Except for the washing solutions, the model is adapted accordingly for a valid comparison of simulative and experimental results. The purification progress is illustrated in Figure 8, while Table 7 provides process yields from process simulation, laboratory-scale experiments and scale-up. Critical process parameters such as pH, ITA concentration and purity can be found in Table 8. For each purification step, results from scale-up are compared to simulation and laboratory-scale experiments.

**Figure A4:**
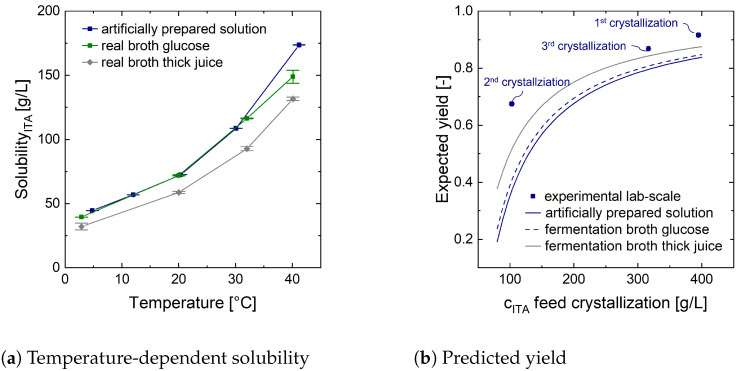
(**a**) Temperature-dependent solubility of fully protonated ITA in water, fermentation broth with glucose and fermentation broth with thick juice. Fermentation broth with glucose is prepared according to Tehrani et al. (2019) [18] with an ammonium chloride concentration of 4 g/L at pH 3.6, fermentation broth with thick juice is obtained from Section 3.2.1. (**b**) Calculated yield depending on ITA concentration in the feed for systems shown in (**a**) correlated with actually obtained yields in laboratory-scale.

In the first cooling crystallization, ITA is concentrated to 348.85 ± 2.71 g/L. The subsequent pH shift decreases ITA concentration to 294.65 ± 4.51 g/L. Crystallization results in a yield of 71.52%, which is considerably lower than 83.49% calculated by process simulations. This can be traced back to the pH shift, which requires 34% more HCl (30 wt%) than suggested by simulation, thereby diluting the solution for crystallization and reducing yield (Figure A4b). Beyond that, the lower yield in scale-up is caused by washing solutions (despite previous saturation with ITA), residuals in piping and losses due to filling and draining of vessels. The increased addition of HCl is also observed in laboratory-scale experiments (Section 3.2.3). However, due to a higher ITA concentration before acid addition, ITA concentration is reduced to 303.78 g/L and the reduced solubility in ITA complex systems can counteract this effect.

In the subsequent cooling crystallization of the mother liquor, the liquid fraction is concentrated to 210.55 ± 4.36 g/L. At this concentration, the solubility limit of NaCl provided by Table 1 (5.27 mol/L) should not be exceeded during the crystallization of the mother liquor. However, it appears that the theoretical solubility of the co-salt is strongly reduced by side components, which is also observed for ITA. As a result, the mother liquor leaving the crystallizer contains only 3.49 mol/L Cl^−^. Inevitably, a co-crystallization occurs, reducing the crystal purity of the second solid fraction to approx. 31%. The co-crystallization also reduces the yield from 75.15%, as calculated in process simulations, to 57.15%. In laboratory-scale, ITA is only concentrated to 102.90 ± 0.15 g/L, avoiding co-crystallization. As the lower concentration is counteracted by decreased ITA solubility in complex systems, the yield loss in laboratory-scale compared to simulation is only small.

Decolorization in scale-up is performed after the dissolution of the solid fractions obtained from the first cooling crystallization and the crystallization of its mother liquor. Compared to laboratory-scale experiments, a high ITA yield of 98.41% is attained. As shown in Figure 8, the broth fed to the evaporative crystallization is very clear and darkens slightly after concentration. A yield of 87.93% corresponds well with process simulations and laboratory-scale experiments. The recovered ITA crystals show a high purity (Table 8) and are optically indistinguishable from commercially acquired ITA crystals.

In future works, scale-up should be performed using Mg(OH)_2_ as base in the fermentation allowing for a higher co-salt solubility and an overall higher ITA yield in the DSP. Additionally, the mother liquor from the final crystallization should be recycled to the first cooling crystallization to evaluate the accumulation of salts in the process and their influence on yield and crystal purity.

## 4. Conclusions and Outlook

A holistic approach to bioprocess development with crude substrates is presented using ITA production with *U. cynodontis* ITA Max pH as an example. A conceptual process design based on data from artificially prepared solutions and fermentations on glucose is used to guide experimental research and to identify main cost drivers early in process development. Over half of the production costs can be allocated to substrate costs, thus the yield shows the highest impact on process economics. By aligning pH-adjusting agents in fermentation and DSP, the process yield can be improved. Despite these insights from simulation, experimental data obtained with real systems is essential to adapt the process to the crude substrate, e.g., by developing a decolorization protocol. Furthermore, multiple carbon sources in the crude substrate prohibit the implementation of a linear feeding profile in the production phase. In this case, feeding controlled by Raman-based online analytics can provide a viable solution for process control. In future works, information obtained from experiments with real systems needs to be implemented into the conceptual process design. This process development strategy, as well as the central experimental findings of this paper, can be transferred to other, more diverse substrates such as molasses or wheat hydrolysates and new products. However, thick juice only shows comparatively few impurities. New challenges resulting from additional side compounds are to be expected.

## Figures and Tables

**Table 1:** Solubility of common co-salts in water at 25 °C.

Co-Salt ^1^	Solubility [72] [g_Anhydrous_/kg_H_2_O_]	Volumetric Solubility ^2^ [mol/L_Solution_]	Volumetric ITA-eq. Solubility [mol_ITA-eq_/L_Solution_]
NaCl	360.00	5.27	2.63
Na_2_SO_4_	281.00	1.79	1.79
NaH_2_PO_4_	949.00	5.63	2.81
NaNO_3_	912.00	7.63	3.81
KCl	355.00	4.03	2.02
K_2_SO_4_	120.00	0.66	0.66
KH_2_PO_4_	250.00	1.66	0.83
KNO_3_	383.00	3.20	1.60
NH_4_Cl	395.00	5.85	2.92
(NH_4_)_2_SO_4_	764.00	4.03	4.03
NH_4_H_2_PO_4_	404.00	2.86	1.43
NH_4_NO_3_	2130.00	11.87	5.94
CaCl_2_	813.00	5.30	5.30
CaSO_4_	2.05	0.02	0.02
Ca(H_2_PO_4_)_2_	No value found	-	-
Ca(NO_3_)_2_	1440.00	5.56	5.56
MgCl_2_	560.00	4.73	4.73
MgSO_4_	357.00	2.61	2.61
Mg(H_2_PO_4_)_2_	No value found	-	-
Mg(NO_3_)_2_	712.00	3.66	3.66

^1^ Chemical formulas are written in anhydrous form, although the actual precipitate may be a hydrate. ^2^ Volume of mixture is calculated ideally.

**Table 2:** Costs of selected acids for pH adjustment before crystallization.

Acid	Specific Acid Price ^1^ [USD/tonne]	Molar Acid Price [USD/kmol]	Number of Protons Provided (pH = 2–7)	Acid Price Per Proton eq. [USD/kmol_H^+^-eq_]
HCl	200	7.29	1	7.29
H_2_SO_4_	200	19.62	2	9.81
H_3_PO_4_	700	68.60	1	68.60
HNO_3_	330	20.79	1	20.79

^1^ Prices are averaged on the grounds of personal communication with suppliers for the German market in 2020.

**Table 3:** Solubility of ITA-salts in water at T = 25 °C and pH > 7 ^1^.

Itaconate Salt	Specific Solubility [g/L]	Molar Solubility [mol/L]
Na_2_ITA	420.18 ± 1.34	2.41 ± 0.01
K_2_ITA ^1^	571.37 ± 56.64	4.39 ± 0.43
MgITA	403.35 ± 13.49	2.65 ± 0.09
CaITA	6.27 ± 0.21	0.04 ± 0.00

^1^ Solubility might be higher, the experiment is terminated at this concentration due to high viscosity.

**Table 4:** Fermentation data of *Ustilago* sp. on pure glucose in pulsed fed-batch fermentations.

Organism	Fermentation Conditions	Product Titer [g_ITA_/L]	Overall Yield [g_ITA_/g_Glucose_]
*U. cynodontis* NBRC 9727 Δfuz7 Δcyp3 P *_etef_mttA* P*_ria1_ria1*	pH = 6.0 (growth phase), pH = 3.6 (production phase), 0.8 g_NH_4_Cl_/L [18]	41.8 ± 0.3	0.39 ± 0.0
*U. maydis* MB215 Δcyp3 ΔMEL ΔUA Δdgat ΔP _*ria*_::P*_etef_* Δfuz7 P*_etef_ mttA*_K14	pH = 6.5, 0.8 g_NH_4_Cl_/L [19]	59.6 ± 5.9	0.42 ± 0.02

**Table 5:** Decolorization resins used in screening experiments.

Decolorization Agent	Abbreviation	Adsorption Behavior	Functional Group	Particle Size [mm]
Activated vegetal charcoal	AC-1	hydrophobic	multiple	powder
Carbon powder activated	AC-2	hydrophobic	multiple	powder
Amberlite XAD-1180	XAD-1180	hydrophobic	none	0.350–0.600
Amberlite IRA-96	IRA-96	weak base anion	tertiary amine ^1^	0.550–0.750
Amberlite IRA-900 (Cl)	IRA-900	strong base anion	trimethylammonium	0.650–0.820
Amberlite IRA-410 (Cl)	IRA-410	strong base anion	dimethylethanol ammonium	0.600-0.750

^1^ no specific data to the nature of tertiary amine available in DuPont datasheet.

**Table 6:** Fermentation KPIs in laboratory-scale and 100 L scale.

Fermentation	ITA Production Normalized to CDW [g_ITA_/g_CDW_]	STY Normalized to CDW [g_ITA_/(h·CDW)]	Yield [g_ITA_/g_glucose_]	Final Titer [g_ITA_/L]
Thick juice, 1L	2.45	0.015	0.46	66.6
Thick juice, 100 L	5.49	0.029	0.44	66.4

**Table 7:** Experimental and simulated ITA yields of each process step.

Process Step	Yield_lab_ [%]	Yield_100L_ [%]	Yield_sim_ [%]
1. concentration and cooling crystallization	91.62	71.52	83.49
2. concentration and cooling crystallization	67.50	57.15	74.15
Decolorization	93.65	98.41	100.00
Evaporative crystallization	86.83	87.93	90.01

**Table 8:** Experimental ^1^ and simulated process variables.

Process Stream	pH_lab_ [-]	pH_100L_ [-]	pH_sim_ [-]	c_ITA,lab_ [g/L]	c_ITA,100L_ [g/L]	c_ITA,sim_ [g/L]	c_Cl^-^,lab_ [mol/L]	c_Cl^-^,100L_ [mol/L]	c_Cl^-^,sim_ [mol/L]	Purity_lab_ [wt%]	Purity_100L_ [wt%]
Filtered broth	3.67	3.67	3.67	62.68 ± 1.04	53.29 ± 0.36	53.33	0.00	0.00	0.00	-	-
Broth after 1st concentration	3.62	3.63	3.67	394.90 ± 1.18	348.85 ± 2.71	353.48	0.20 ± 0.01	0.00	0.00	-	-
Broth after 1st pH shift	2.80	2.14 ^2^	1.94	303.78 ± 0.94	294.65 ± 4.51	310.09	- ^3^	1.06 ± 0.02	0.94	-	-
1st Solid fraction	-	-	-	-	-	-	-	-	-	97.65 ± 0.01	97.64 ± 0.24
Mother liquor after 1st crystallization	2.83	2.62	2.80	41.30 ± 0.35	59.52 ± 0.22	69.63	1.75 ± 0.01	1.66 ± 0.01	1.21	-	-
Broth after 2nd concentration	2.54	2.27	2.71	102.90 ± 0.15	210.55 ± 4.36	201.16	4.41 ± 0.05	3.12 ± 0.06	3.51	-	-
Broth after 2nd pH shift	2.56	2.27	2.02	102.90 ± 0.15 ^3^	210.55 ± 4.36	198.98	- ^3^	3.12 ± 0.06	3.55	-	-
2nd Solid fraction	-	-	-	-	-	-	-	-	-	101.63 ± 0.59	31.22 ± 4.06
Mother liquor after 2nd crystallization	2.72	2.55	2.80	37.56 ± 0.34	111.15 ± 4.17	67.47	4.74 ± 0.04	3.49 ± 0.12	4.48	-	-
Solution after Dissolution	1.99	1.96	2.06	72.94 ± 0.27	72.54 ± 0.32	72.48	0.06 ± 0.01	0.05 ± 0.00	0.00	-	-
Solution after Decolorization	1.98	2.08	2.06	69.65 ± 0.46	69.95 ± 0.84	72.48	0.06 ± 0.01	0.05 ± 0.00	0.00	-	-
3rd Solid fraction	-	-	-	-	-	-	-	-	-	98.57 ± 3.39	101.05 ± 1.00
Mother liquor after 3rd crystallization	2.14	2.45	2.06	55.02 ± 0.06	49.23 ± 0.83	73.82	0.59 ± 0.01	1.55 ± 0.01	0.00	-	-

^1^ Experimental parameters are determined offline at 20 °C; ^2^ Determined online at approx. 60 °C since ambient conditions cause solid precipitation; ^3^ not determined as pH-shift and cooling are conducted simultaneously (Appendix L).

**Figure A1:**
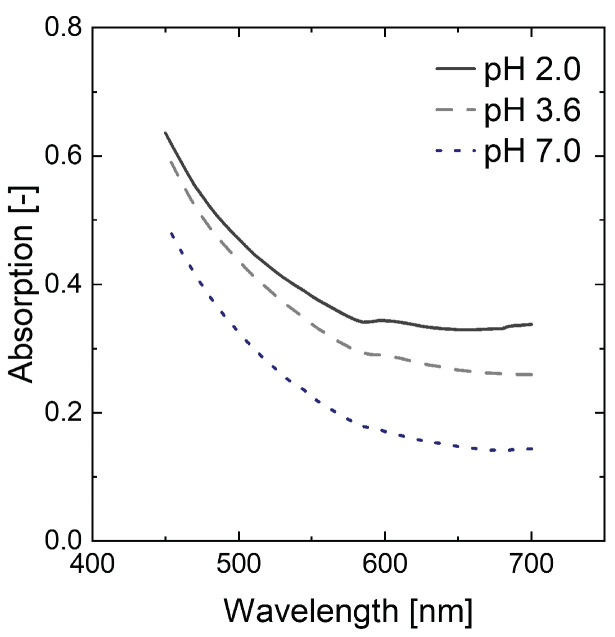
Differences in adsorption from artificially prepared broth depending on pH.

**Figure A2:**
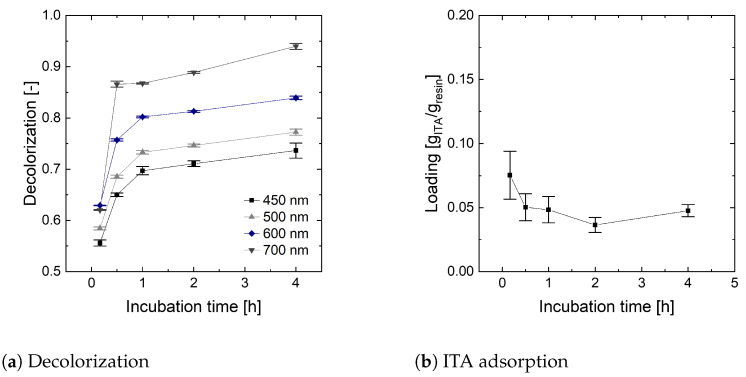
Determination of minimum incubation time for decolorization.

**Figure A3:**
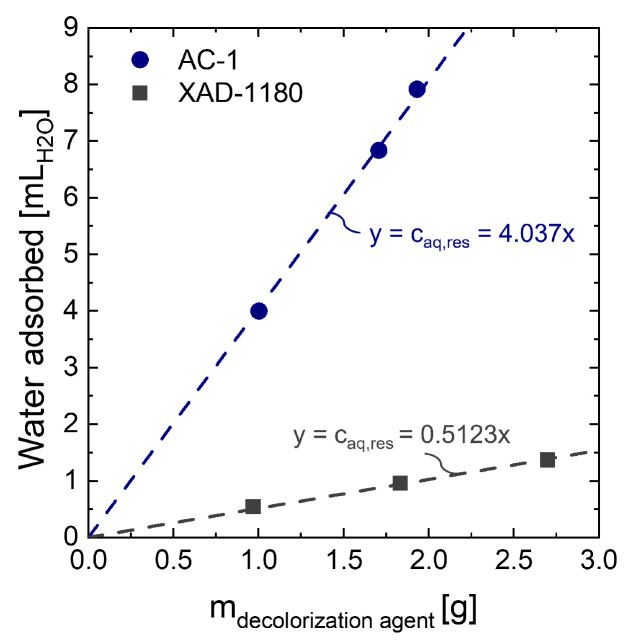
Water retained in XAD-1180 resin and AC-1 after incubation with distilled water.

**Figure A5:**
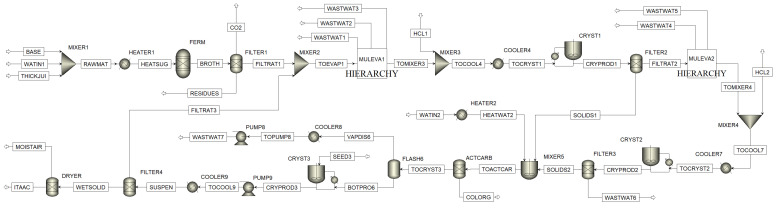
Aspen process flowsheet of multiple crystallization process.

**Figure A6:**
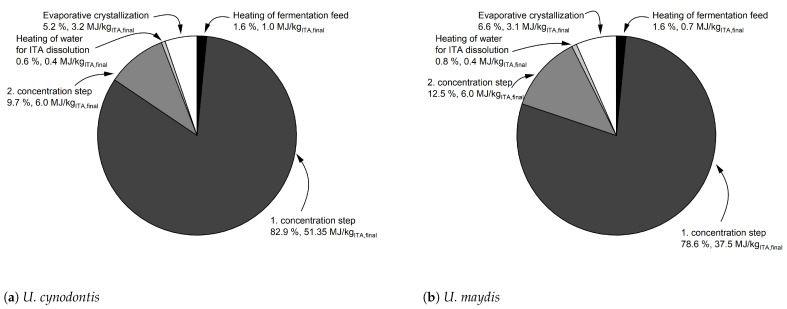
Gross steam requirement (without energy integration measures).

**Figure A7:**
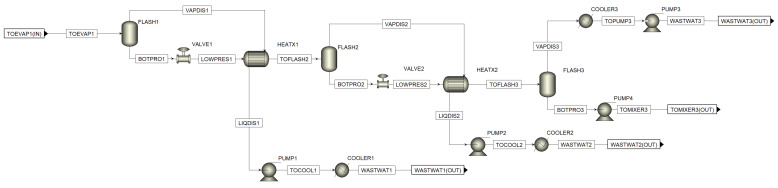
Aspen process flowsheet of MEE in the first cooling crystallization sequence.

**Figure A8:**
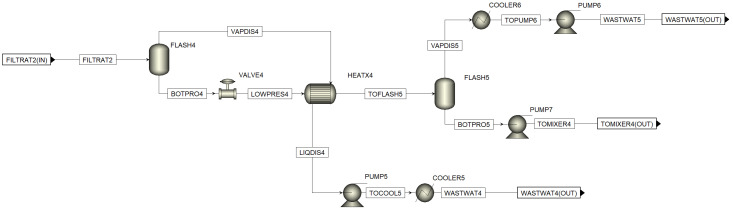
Aspen process flowsheet of MEE in the second cooling crystallization sequence.

**Figure A9:**
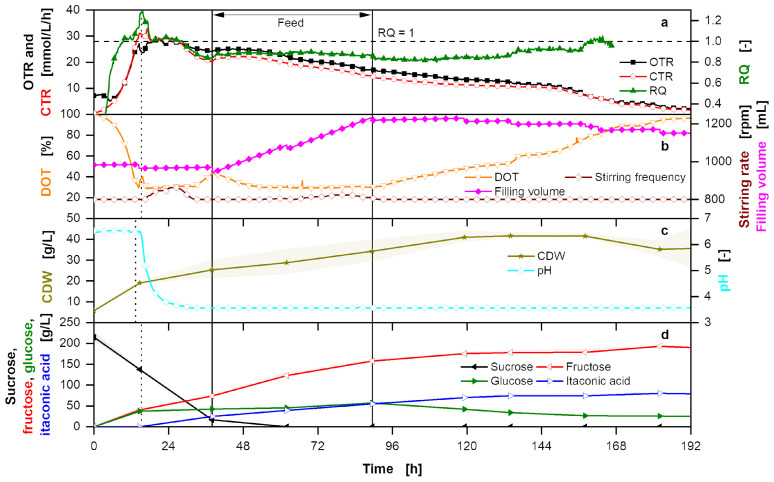
Overfed extended-batch fermentation of *U. cynodontis* ITA Max pH with pH shift and thick juice as carbon source. (**a**) OTR, CTR and RQ. The horizontal dashed line shows RQ = 1. RQ values are only shown for OTR values > 5 mmol/L/h. (**b**) DOT, stirring rate and filling volume. (**c**) CDW and pH. (**d**) Sucrose, glucose, fructose and ITA concentration. For the batch phase, 215 g/L sucrose from thick juice are initially added to the medium. During the feed phase (between the vertical solid lines), 215 g of additional sucrose from thick juice are added into the fermentation vessel. Cultivation is performed in a 2 L stirred tank reactor with an initial filling volume of 1 L at 30 °C with a constant aeration and stirring rate of 1 L/min and 1200 rpm, respectively. For clarity, only every 20th measured online data point is shown.

**Figure A10:**
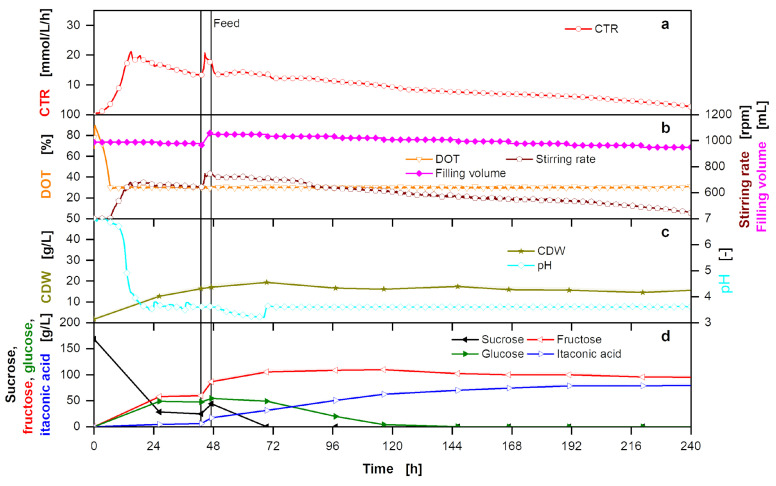
Overfed extended-batch fermentation of *U. cynodontis* ITA Max pH with Mg(OH)_2_ as pH-adjusting agent and thick juice as carbon source. (**a**) CTR. (**b**) DOT, stirring rate and filling volume. (**c**) CDW and pH. (**d**) Sucrose, glucose, fructose and ITA concentration. For the batch phase, 160 g/L sucrose from thick juice are initially added to the medium. During the feed phase (between the vertical solid lines) 100 g of additional sucrose from thick juice are added into the fermentation vessel. Cultivation is performed in a 2 L stirred tank reactor with an initial filling volume of 1 L at 30 °C with a constant aeration rate of 1 L/min. For clarity, only every 20th measured online data point is shown.

**Figure A11:**
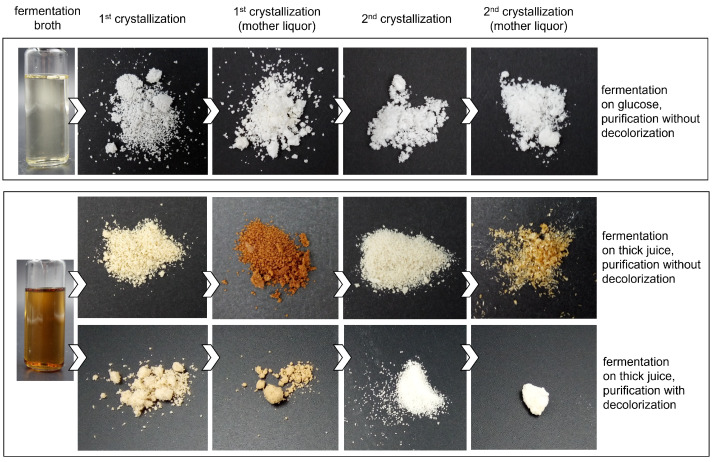
Discolouration of crystals after purification from fermentation broth with thick juice and glucose without decolorization. Fermentation broth on glucose is prepared as described in Tehrani et al. (2019) [18].

**Figure A12:**
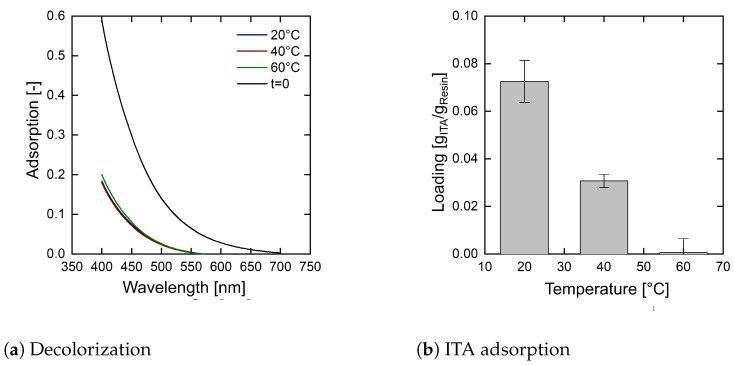
(**a**) Temperature-dependent decolorization behavior of artificial broth at a starting pH of 3.6. (**b**) Temperature-dependent adsorption behavior of ITA in artificial broth at a starting pH of 3.6.

**Figure A13:**
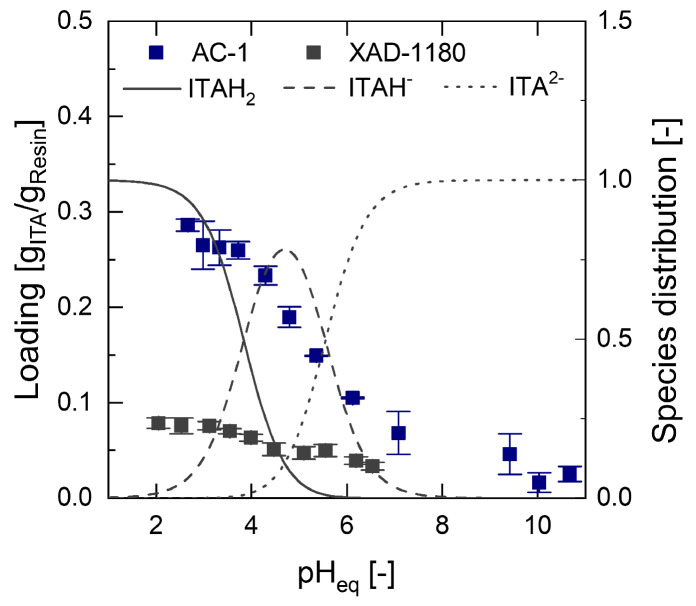
Adsorption of ITA on AC-1 and XAD-1180 at different pH values in artificially prepared systems and respective species distribution.

**Figure A14:**
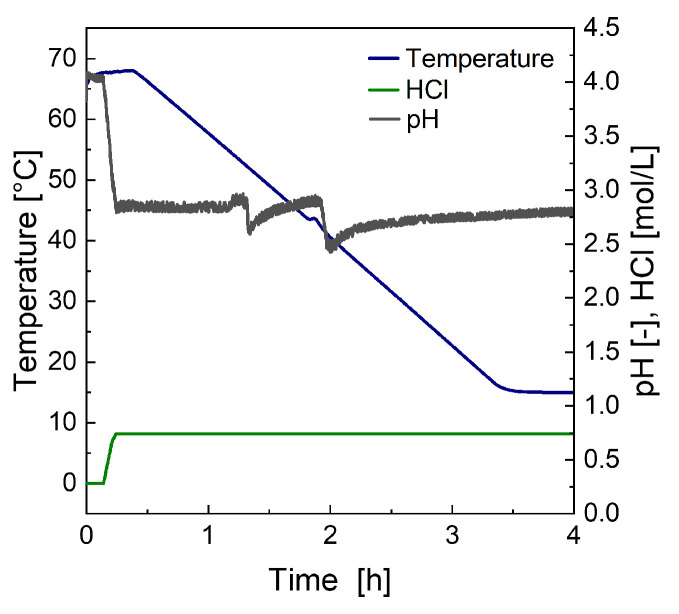
Exemplary crystallization in laboratory-scale.

**Figure A15:**
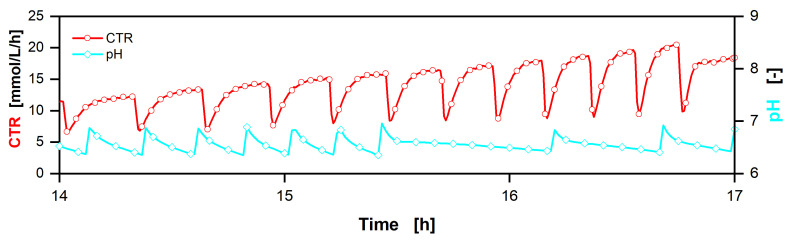
Close-up view of pH and CTR in the 100 L batch fermentation, shown in Figure 6, before the pH shift. Cultivation is performed in a 150 L stirred tank pressure reactor with an initial filling volume of 105 L at 30 °C with a constant stirring rate of 285 rpm. For clarity, only every fifth measured online data point is shown.

**Figure A16:**
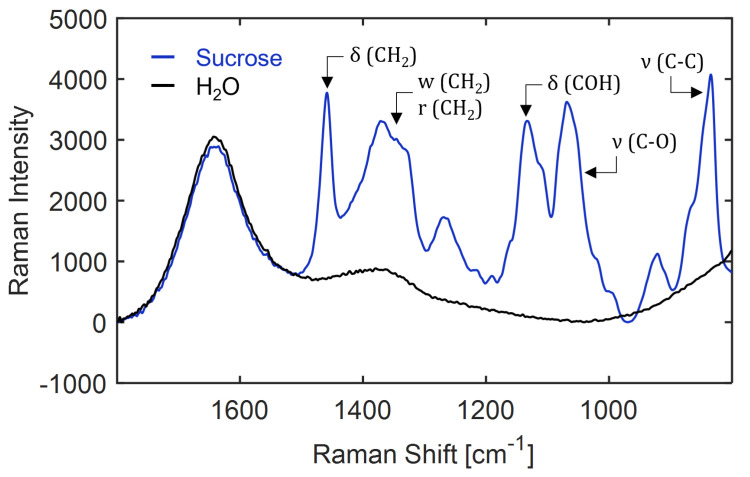
Assignment of molecular vibrations to the Raman spectrum of 10 wt% sucrose in water recorded at 25 ${}^{\circ }$C. The pure water spectrum (black curve) is shown for reference. ν: stretching, δ: bending, *w*: wagging, *r*: rocking and γ: deformation modes.

**Figure A17:**
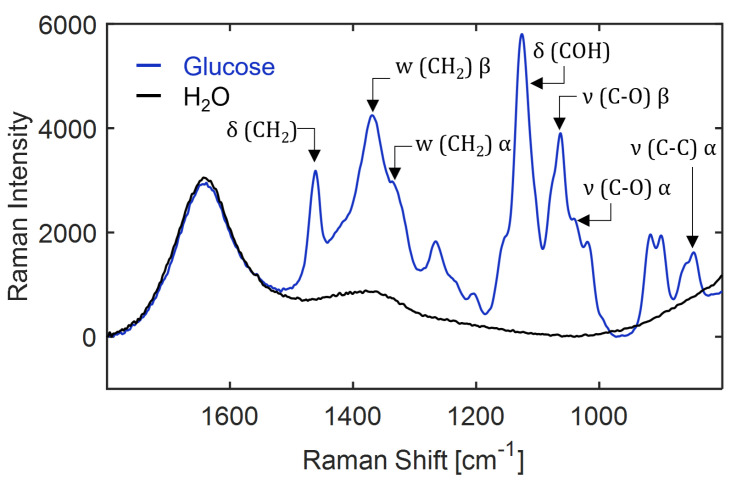
Assignment of molecular vibrations to the Raman spectrum of 10 wt% glucose in water recorded at 25 ${}^{\circ }$C. The pure water spectrum (black curve) is shown for reference. ν: stretching, δ: bending and *w*: wagging modes, α: alpha-D-glucose, β: beta-D-glucose.

**Figure A18:**
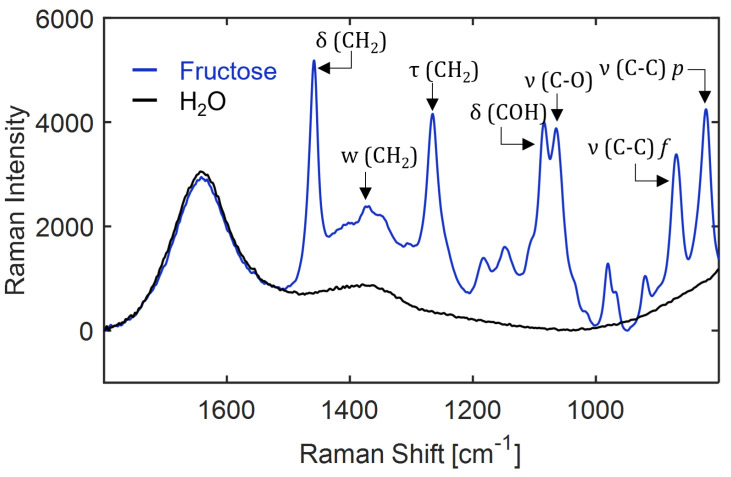
Assignment of molecular vibrations to the Raman spectrum of 10 wt% fructose in water recorded at 25 ${}^{\circ }$C. The pure water spectrum (black curve) is shown for reference. ν: stretching, δ: bending, *w*: wagging and τ: twisting modes, *f*: D-fructofuranose, *p*: D-fructopyranose.

**Figure A19:**
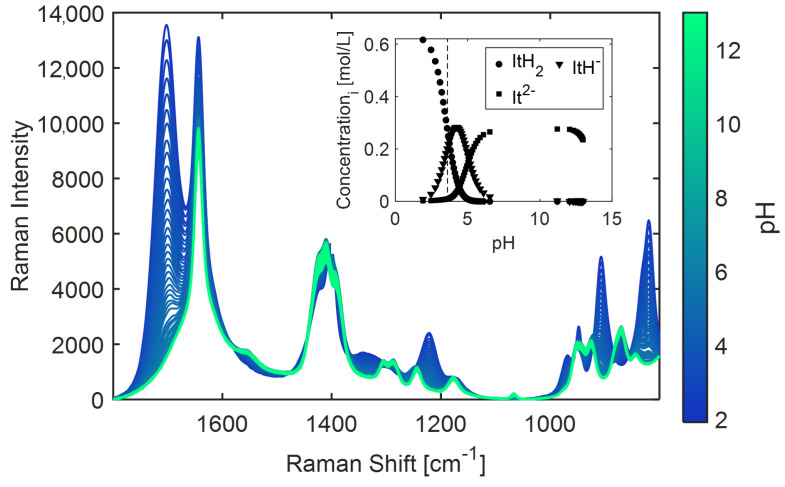
Raman spectra recorded during titration of 8 wt% ITA with 1 M NaOH solution at 25 ${}^{\circ }$C. Spectra are treated with a linear baseline. Inset shows the concentration profiles of the ITA species, calculated using non-ideal thermodynamics, as in Echtermeyer et al. (2021) [93]. ITAH_2_: undissociated, ITAH^−^: first dissociation state and ITA^2−^: fully dissociated ITA.

**Figure A20:**
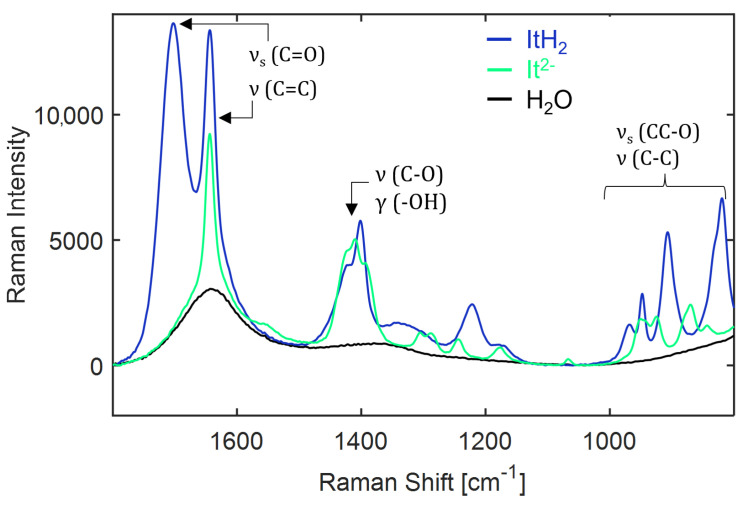
Assignment of molecular vibrations to the Raman spectrum of 8 wt% (ITAH_2_) and 3 wt% (ITA^2−^) ITA in water recorded at 25 ${}^{\circ }$C. The pure water spectrum (black curve) is shown for reference. ν: stretching, $\nu_{s}$: antisymmetric stretching and γ: deformation modes. ITAH_2_: undissociated and ITA^2−^: fully dissociated ITA.

**Figure A21:**
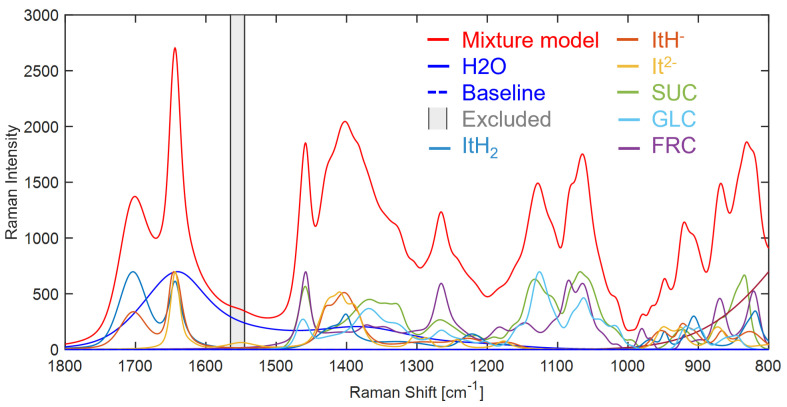
IHM constructed for ITA dissociation with sucrose, glucose and fructose and water solvent. ITAH_2_: undissociated, ITAH^−^: first dissociation state and ITA^2−^: fully dissociated ITA, SUC: sucrose, GLC: glucose, FRC: fructose.

**Figure A22:**
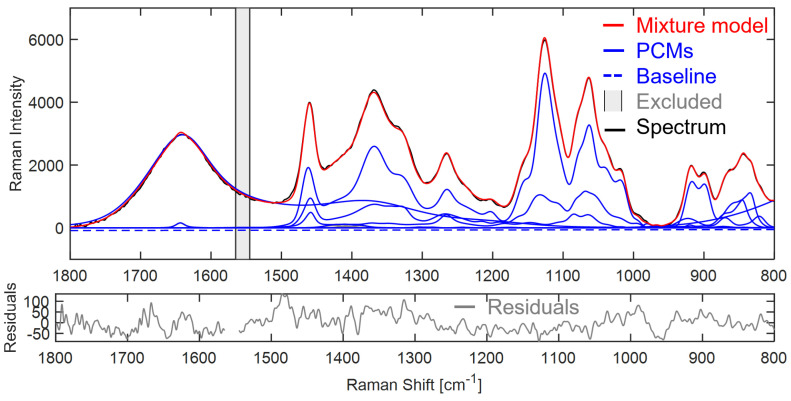
The mixture hard model consisting of sucrose, glucose, fructose and ITA dissociation PCMs fitted to a Raman spectrum of 1 wt% fructose, 3 wt% sucrose and 8 wt% glucose. It has a linear baseline and excluded range between 1545 and 1565 cm^−1^.

**Figure A23:**
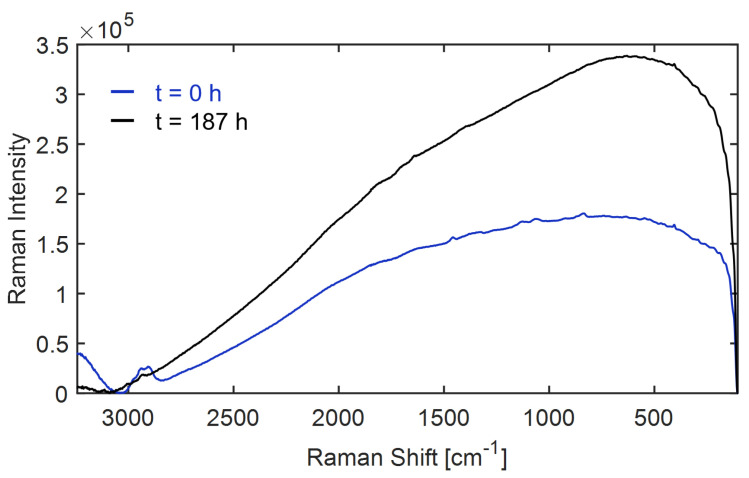
Raman spectra recorded inline at the start (t = 0 h) and end (t = 187 h) during the 100 L fermentation.

## Data Availability

All data concerning this manuscript are avaliable from the corresponding author upon reasonable request.

## Abbreviations

The following abbreviations are used in this manuscript:

CDW	Cell Dry Weight
CFA	Confirmatory Factor Analysis
CTR	CO_2_ Transfer Rate
DOT	Dissolved Oxygen Tension
DSP	Downstream Processing
GC-ToF-MS	Gas Chromatography Time-of-Flight Mass Spectrometry
glucose eq.	Glucose equivalent of thick juice
HPLC	High Performance Liquid Chromatography
IC	Ion Exchange Chromatography
ICP-OES	Inductively Coupled Plasma Optical Emission spectroscopy
IHM	Indirect Hard Modelling
ITA	Itaconic Acid
ITAH_2_	Fully protonated itaconic acid
ITAH^−^	Single deprotonated itaconic acid
ITA^2−^	Fully deprotonated itaconic acid
KPI	Key Performance Indicators
LOD_i_	Limit of Detection
OTR	O_2_ Transfer Rate
OD_600_	Optical Density at 600 nm
RMSEC_i_	Root mean squared error of cross-validation
RMSECV_i_	Root mean squared error of leave-10% cross-validation
RQ	Respiratory Quotient
STY	Space-Time Yield
Y	Yield

## Appendix A. Substrate Analysis

Substrate analysis is performed using gas chromatography time-of-flight mass spectrometry (GC-ToF-MS) as published in de Wit et al. (2022) [96] for qualitative analysis of impurities. For quantitative analysis, inductively coupled plasma optical emission spectroscopy (ICP-OES) is used for cations while anions are quantified via ion exchange chromatography (IC). Additionally, NH_4_^+^ and PO_4_^3−^ concentration are determined by confirmatory factor analysis (CFA). HPLC according to Section 2.2.4 is used with a column of 30 cm length, 8 mm in diameter at a flowrate of 0.6 mL/min to quantify sugar and lactic acid content. The resulting data is depicted in Table A1. Density is determined to 1.3310 ± 0.0023 g/cm³ with a DMA48 density meter (Anton Paar, Graz, Austria) at 20 °C. Even though pigments are not detected using GC-ToF-MS, ICP-OES, IC, CFA or HPLC, thick juice shows a light to medium brown color. As it can be found in the literature, colorization of thick juice occurs during sugar production from the alkaline degradation of hexose sugars and Maillard reactions forming melanoidins [83]. Both compound groups have a large molecule size of over 2 kDa [83] and contain a variety of active groups such as carboxyl and aldehyde groups [83,84]. Furthermore, colourants not related to sucrose, e.g., phenols, flavonoids and melanin are present in crude substrates [78]. In both cases, due to their size and active groups, most of these molecules are expected to be of a more hydrophobic nature at low pH values.

**Table A1:** Composition of thick juice.

Analyte	Measurement	Unit	Method
Sucrose	842 ± 8	g/L	HPLC
Glucose	>1	g/L	HPLC
Fructose	>1	g/L	HPLC
Lactic acid	1.17 ± 0.13	g/L	HPLC
Cl^−^	0.048 ± 0.000	wt%	IC
NO_3_^−^	0.0524 ± 0.01	wt%	IC
SO_4_^2−^	0.0833 ± 0.01	wt%	IC
Ca	72.93 ± 1.00	mg/kg	ICP-OES
Fe	<2	mg/kg	ICP-OES
K	6660 ± 20	mg/kg	ICP-OES
Mg	12.11 ± 0.16	mg/kg	ICP-OES
Na	547 ± 3	mg/kg	ICP-OES
P	35 ± 3	mg/kg	ICP-OES
NH_4_^+^	0.0122	wt%	CFA
PO_3_^3−^	>0.0008	wt%	CFA

## Appendix B. Calculations of Yield, STY, CTR, OTR and RQ for Fermentation Evaluation

To evaluate fermentative performance, yield, STY, CTR, OTR and RQ are calculated based on mass balances. Equation (A1) shows how to calculate the mass balance for a compound from fermentation.

(A1) $$m_{x}\left(t\right)=c_{x}\left(t_{0}\right)\cdot V_{L}\left(t_{0}\right)+c_{x,feed}\cdot V_{feed}\left(t\right)-\sum_{t_{0}}^{t}c_{x}\left(t\right)\cdot V_{sample}\left(t\right)$$

With $m_{x}\left(t\right)$ [g] as the calculated mass at time point *t*, $c_{x}$($t_{0}$) [g/L] as the concentration of *x* at the beginning of the fermentation, $V_{L}$(t${}_{0}$) [L] as the initial filling volume, $c_{x,feed}$ [g/L] as the concentration of *x* in the feed and $V_{feed}\left(t\right)$ [L] as the added feeding volume at time *t*. Sampling is considered by summing up the substrate concentration $c_{x}$(t) [g/L] times the corresponding sample volume $V_{sample}\left(t\right)$ [L].

The yield Y(t) [g_ITA_/g_substrate_] can be calculated by dividing the mass of ITA produced by the summed up mass of the substrates glucose, fructose and sucrose (Equation (A2)).

(A2) $$Y\left(t\right)=\frac{m_{ITA}\left(t\right)}{\left(m_{glucose}\left(t\right)+m_{fructose}\left(t\right)+m_{sucrose}\left(t\right)\right)}$$

STY [g_ITA_/(L·h)] is calculated by dividing the mass of ITA $m_{ITA}$ [g] by the sum of the filling volume $V_{L}$ [L] of the fermenter and the total sample volume $V_{sample}\left(t\right)$ [L] times the fermentation time *t* [h] (Equation (A3)).

(A3) $$STY\left(t\right)=\frac{m_{ITA}\left(t\right)}{(V_{L}\left(t\right)+V_{sample}\left(t\right))\cdot t}$$

Equations (A4) and (A5) describe the calculation of CTR and OTR using the gas flow rate into the fermenter $q_{in}$ [L/h], the fermentation volume $V_{L}$ [L], the molar volume of an ideal gas $V_{M}$ [L/mmol] at 25 °C and the O_2_ and CO_2_ fractions in the inlet and outlet $y_{O2,\mathrm{in}}$, $y_{O2,\mathrm{out}}$, $y_{CO2,in}$ and $y_{CO2,\mathrm{out}}$, [-], respectively.

(A4) $$CTR=\frac{q_{in}}{V_{F}\cdot V_{M}}\cdot \left(y_{CO2,out}\cdot \frac{1-y_{O2,in}-y_{CO2,in}}{1-y_{O2,out}-y_{CO2,out}}-y_{CO2,in}\right)$$

(A5) $$OTR=\frac{q_{in}}{V_{F}\cdot V_{M}}\cdot \left(y_{O2,in}-\frac{1-y_{O2,in}-y_{CO2,in}}{1-y_{O2,out}-y_{CO2,out}}\cdot y_{O2,out}\right)$$

Equation (A6) describes the calculation of the RQ corresponding to CTR and OTR.

(A6) $$RQ=\frac{CTR}{OTR}$$

Equations (A7) and (A8) describe the RQ of growth on pure glucose. Calculations are based on the biomass composition from Klement et al. (2012) [76].

(A7) $$C_{6}H_{12}O_{6}+4.95\cdot O_{2}+0.1\cdot NH_{4}^{+}=CH_{1.8}N_{0.1}O_{0.6}+5\cdot CO_{2}+5.3\cdot H_{2}O$$

(A8) $$RQ=\frac{CTR}{OTR}=\frac{5}{4.95}=1.01$$

Equations (A9) and (A10) describe the theoretical RQ of ITA production on pure glucose.

(A9) $$C_{6}H_{12}O_{6}+1.5\cdot O_{2}=C_{5}H_{6}O_{4}+CO_{2}+3\cdot H_{2}O$$

(A10) $$RQ=\frac{CTR}{OTR}=\frac{1}{1.5}=0.67$$

ITA, sucrose, glucose and fructose concentration and CDW are analyzed in triplicates, mapping the error of pipetting and concentration measurements. Volumes are determined once. From there, fermentation KPIs are calculated and the mean is given. The standard deviation from the mean is also calculated and is below 5% for all values. OTR, CTR, DOT and pH are determined once for the corresponding fermentation time point.

## Appendix C. Establishing a Protocol for Screening of Decolorization Resins

For characterization of the decolorization performance, adsorption is determined between 450 nm and 700 nm. ITA does not absorb light in this wavelength range and therefore does not contribute to the overall signal [97]. When the absorption of the artificially prepared broth prior to decolorization is investigated, no clear peaks can be identified (Figure A1). This suggests multiple compounds being involved in the colorization of the fermentation broth [79,83,84]. Furthermore, a pH dependence in adsorption is observed, indicating the presence of some pH sensitive compounds. As dilution of samples leads to a change in pH and thus adsorption, samples are measured in undiluted form. The remaining 5% of wash water are considered by diluting the reference solution accordingly.

To determine the minimum incubation time, the XAD-1180 resin is used with artificially prepared broth in an exemplary study. As decolorization and ITA adsorption are constant after 4 h incubation time, the minimum incubation time is set accordingly (Figure A2).

To calculate the resin loading with ITA $L_{res}$ [g_ITA_/g_resin_], the remaining volume of washing water $V_{wash}$ [L] is considered in combination with the volume of the solution added for decolorization $V_{add.}$ [L], the ITA concentration before $c_{t=0}$ and after incubation $c_{t=1}$ and the amount of resin used $m_{res}$ [g]. (Equation (A11)).

(A11) $$L_{res}=\frac{V_{F,c.art.}\cdot c_{t=0}-\left(V_{add}+V_{wash}\right)\cdot c_{t=1}}{m_{resin}}$$

For yield calculations in decolorization depending on resin concentration, the amount of liquid remaining in decolorization agents after filtration and therefore contributing to yield loss is considered. Otherwise, process yield is overestimated. To determine the amount of liquid bound in the decolorization agent after filtration, different amounts of decolorization agent are incubated with water similar to the washing step in the screening experiments mentioned above. After vacuum filtration, the recovered water is quantified. To subtract the amount of water lost in the filter, the same filtration protocol is conducted with water only. In each filter, 4.0 ± 0.1 g distilled water are incorporated.

Yield calculation with yield loss due to water $Y_{ITA,c}$ is performed using the amount of water lost per g resin $c_{aq,res}$, which can be deduced from the slope of the linear function in Figure A3 (Equation (A12)).

(A12) $$Y_{ITA,c}=\frac{c_{ITA,t=1}\cdot \left(V_{wash}+\left(V_{add}-c_{aq,res}\cdot m_{res}\right)\right)}{V_{add}\cdot c_{ITA,t=0}}$$

## Appendix D. Calculation of Yield and Purity for Downstream Process Evaluation

A yield calculation for each unit operation is performed by dividing the mass of ITA after the unit operation $t_{n+1}$ [g] by the mass of ITA before the unit operation $t_{n}$ [g] to close the mass balance for each unit operation. The mass of ITA is calculated from the concentration $c_{ITA}$ [g/L], the density of the aqueous phase $\rho_{aq}$ [g/L] and the mass of the aqueous phase $m_{aq}$ [g] (Equation (A13)). The overall process yield was calculated based on the amount of ITA after cell separation. The standard deviations reflect errors in HPLC measurements.

(A13) $$Y_{ITA}=\frac{c_{ITA}\left(t_{n+1}\right)\cdot \frac{m_{aq}\left(t_{n+1}\right)}{\rho_{aq}\left(t_{n+1}\right)}}{c_{ITA}\left(t_{n}\right)\cdot \frac{m_{aq}\left(t_{n}\right)}{\rho_{aq}\left(t_{n}\right)}}$$

Purity of ITA, $P_{ITA}$ [-], is calculated dividing the ITA concentration by the gravimetrically determined crystal concentration $c_{c,grav}$ [g/L].

(A14) $$P_{ITA}=\frac{c_{ITA,HPLC}}{c_{c,grav}}$$

Similar to fermentation, ITA, sucrose, glucose and fructose concentration are analyzed in triplicates, mapping the error of pipetting and concentration measurements. Absorption at different wavelengths and volumes are determined once. From there, DSP KPIs are calculated and the mean is given. The standard deviation from the mean is also calculated and is below 5% for all values.

## Appendix E. Temperature-Dependent Solubility of Fully Protonated ITA in Pure and Complex Systems

For process modeling in Aspen Plus, solubility of fully protonated ITA is determined in distilled water. To be able to interpret experimental data with complex systems, solubility is evaluated in fermentation broth with glucose and thick juice (Figure A12).

## Appendix F. Process Modeling in Aspen Plus and Techno-Economic Analysis

The flowsheet model is set up in Aspen Plus and displayed in Figure A5. ELECNRTL is used as global property method. UNIFAC is applied to determine missing binary interaction parameters. The Elec Wizard allowed the implementation of dissociation and precipitation behavior of inorganic components. Deprotonated ITA species are not available in Aspen component databanks. Thus, they are added by molecular structure. Respective dissociation reactions are set up in the global chemistry environment. For crystallization blocks, the method is locally adapted to SOLIDS.

The feed is modeled as glucose–water mixture representing the typical sugar content of thick juice (0.65 wt%). The water feed and base mass flows are defined by product titer and pH during product formation, respectively, (Table 4). The fermenter is implemented as RYIELD modeling the reaction as de-lumping of glucose to ITA and CO_2_. The overall yields in Table 4 are used as reaction coefficients. A full consumption of the sugar content is assumed. CO_2_ is isolated from the broth in a separator model. RESIDUES contains the residual sugar content that is not converted to ITA or CO_2_ and would in a real fermentation be used for biomass formation or maintenance metabolism. Additional nutrients such as NH_4_Cl are not considered in the simulation.

Steam is identified as the major energy cost driver in the presented process concept (Figure 2). Figure A6 displays the steam consuming process steps. The evaporation steps in the first and second cooling crystallization sequence require the largest fractions of the total steam demand. Thus, we implement multiple-effect evaporators (MEE) for those concentration units to mitigate the energy requirement of the proposed process. For the purpose of flowsheet modeling, a three-stage and a two-stage MEE are implemented in the first and second cooling crystallization sequence reducing steam requirement by approximately 60% and 40% in comparison to simple flash evaporators, respectively. The pressure drop in each throttle is adjusted to just meet the minimum temperature requirement of 10 K in the heat exchangers. As described in Section 3.1.1, in the first MEE the final stage concentrates the broth up to a concentration of 350 g_ITA_/L. However, in the second MEE the broth is concentrated so that the solubility limit of the co-salt MgCl_2_ is just not exceeded during the subsequent crystallization. That way, overall ITA yield is maximized. The Aspen flowsheets for the MEEs used in this process flowsheet are displayed in Figure A7 and Figure A8.

The cooling crystallization steps lower the temperature to 15 °C at 1 bar. The effluents are at solid–liquid equilibrium. The according data is experimentally determined with artificially prepared ITA systems (Figure A4a) and supplied to the model. After the second cooling crystallization sequence only the liquid fraction contains the co-salt which is then to be disposed. The saline mass fraction of this stream is priced with 110 EUR/tonne (Table A2). The solid fractions of the cooling crystallization sequences are mixed. Just enough water is added to the system until all solids are dissolved at 80 °C. The elevated temperature limits the amount of water to be evaporated in succeeding process steps. Afterwards the system is subject to an activated carbon treatment, which is only symbolically added to the Aspen flowsheet. Thus, the COLORG stream is a zero-flow. The stream is then fed to the evaporative crystallization which is operated at 80 °C. To reach an ITA yield of 90% after the crystallization, the vacuum pressure is selected accordingly. The seeds added amount to 5 wt% of the solids produced by the crystallizer. After solid–liquid separation, the mother liquor is recycled to the beginning of the DSP. Similar to the activated carbon adsorber, a dryer for ITA crystals is added to the flowsheet for symbolic reasons. Thus, MOISTAIR stream is a zero-flow.

To retrieve heat/cooling duties from crystallizers in Aspen Plus poses difficulties. Thus, coolers and a flash evaporator are integrated into the flowsheet before the cooling and the evaporative crystallizer, respectively. The operating parameters of the coolers and the flash evaporator are aligned with the crystallizer to obtain the duties required by the crystallization steps. Since ITA crystallization only occurs in the crystallizers, the energetic duty resulting from crystal formation is calculated separately using the mass flow of crystal product and the enthalpy of fusion for ITA ($\Delta H_{\mathrm{fus}}(171.3{\;}^{\circ }$C) = 32.2 kJ/mol [98]).

All solid–liquid phase separations in the process are calculated ideally. Pumps are only integrated in the flowsheet when an elevated pressure has to be generated in a liquid phase. Coolers operate nearly isobaric. Waste water streams are cooled down by air and leave the system at ambient pressure.

As the simulation is solely applied for an operative cost analysis, the mass flows are scaled with respect to the amount of ITA produced by the process. Stream and utility prices used for the operative cost analysis are displayed in Table A2. Detailed parameterization for *U. cynodontis* ITA Max pH and *U. maydis* K14 simulation can be retrieved from Table A3.

**Table A2:** Stream, waste and utility prices.

Stream/Utility	Composition	Price	Unit	Reference
Thick juice	65 wt%	211.54	EUR/tonne	^1^
Mg(OH)_2_	40 wt%	100.00	EUR/tonne	^2^
HCl	30 wt%	80.00	EUR/tonne	^2^
Process water	100 wt%	1.40	EUR/tonne	^2^
Waste water	-	2.90	EUR/tonne	^2^
Saline waste water	-	110	EUR/tonne	^2,3^
Electricity	-	0.0849	EUR/kWh	[99]
Low pressure steam	-	27.00	EUR/tonne	^2^
Cooling water	-	0.05	EUR/kWh	^2,4^
Process and utility-related CO_2_ emissions	-	27.37	EUR/tonne	[100]

^1^ USD 374.27 /tonne_Sugar_ (07.2020, CIF European ports), USD/EUR 1.15 (Exchange rate 07.2020 [101]), Thick juice price derived with respect to sugar weight fraction. ^2^ Price averaged on the grounds of personal communication with suppliers and industry representatives for the German market in 2020. ^3^ The provided price is only used for the saline mass fraction of WASTWAT6 since it contains large fractions of MgCl_2_. The regular waste water price is applied to the water mass fraction of WASTWAT6. ^4^ Moderately low temperature refrigerated water (Inlet: T = 5 °C, 1 bar).

**Table A3:** Parameterization of *U. cynodontis* ITA Max pH and *U. maydis* K14 simulations.

Parameter	*U. cynodontis* ITA Max pH	*U. maydis* K14
Thick juice mass flow	3.84 kg/kg_ITA,final_	3.95 kg/kg_ITA,final_
Water feed mass flow	20.94 kg/kg_ITA,final_	14.76 kg/kg_ITA,final_
Base mass flow	0.12 kg/kg_ITA,final_	0.70 kg/kg_ITA,final_
HEATER1 Temperature	35.00 °C	35.00 °C
HEATER1 Outlet pressure	1.00 bar	1.00 bar
De-lumping coefficient ITA	0.39 g_ITA_/g_glucose_	0.42 g_ITA_/g_glucose_
De-lumping coefficient CO_2_	0.1323 g_CO_2__/g_glucose_	0.1425 g_CO_2__/g_glucose_
FLASH1 Temperature	100.00 °C	100.00 °C
FLASH1 Vapor mass fraction	0.27	0.25
VALVE1 Outlet pressure	0.59 bar	0.57 bar
HEATX1 Hot stream outlet temperature	97.00 K	97.00 K
HEATX1 ΔT_min_	10.00 K	10.00 K
FLASH2 Temperature	86.00 °C	87.00 °C
FLASH2 Vapor mass fraction	0.39	0.35
VALVE2 Outlet pressure	0.33 bar	0.30 bar
HEATX2 Hot stream outlet temperature	82.00 K	82.00 K
HEATX2 ΔT_min_	10.00 K	10.00 K
FLASH3 Temperature	72.28 °C	76.20 °C
FLASH3 Vapor mass fraction	0.67	0.56
HCl (1. cooling crystallization)	0.33 kg/kg_ITA_	1.93 kg/kg_ITA_
COOLER4 Temperature	15.00 °C	15.00 °C
COOLER4 Pressure	1.00 bar	1.00 bar
CRYST1 Temperature	15.00 °C	15.00 °C
CRYST1 Pressure	1.00 bar	1.00 bar
FLASH4 Temperature	100.00 °C	100.00 °C
FLASH4 Vapor mass fraction	0.39	0.20
VALVE4 Outlet pressure	0.39 bar	0.29 bar
HEATX4 Hot stream outlet temperature	97.00 K	87.00 K
HEATX4 ΔT_min_	10.00 K	10.00 K
FLASH5 Temperature	86.07 °C	85.77 °C
FLASH5 Vapor mass fraction	0.64	0.27
HCl (2. cooling crystallization)	0.007 kg/kg_ITA,final_	0.002 kg/kg_ITA,final_
COOLER7 Temperature	15.00 °C	15.00 °C
COOLER7 Pressure	1.00 bar	1.00 bar
CRYST2 Temperature	15.00 °C	15.00 °C
CRYST2 Pressure	1.00 bar	1.00 bar
Water added for dissolution	1.68 kg/kg_ITA,final_	1.64 kg/kg_ITA,final_
MIXER 5 Temperature	80.00 °C	80.00 °C
MIXER 5 Pressure	1.00 bar	1.00 bar
FLASH6 Temperature	80.00 °C	80.00 °C
FLASH6 Vapor mass fraction	0.55	0.54

## Appendix G. Overfed Fermentation of *U. cynodontis* ITA Max pH

A fermentation with an elongated feed profile with thick juice is depicted in Figure A9. The product yield is low, as not all substrate is used from the fermentation broth. However, this is not due to substrate inhibition. Even with high sugar concentrations of over 200 g/L sucrose, a maximum titer of 80 g/L, similiar to fermentations on glucose [74] and to the fermentation shown in Figure 3, is reached. Therefore, product inhibition by weak organic acid stress [74,89] is responsible for reduced cell viability and prevents further substrate conversion. Furthermore, no limitation in oxygen transfer due to high substrate concentration and high medium osmolarity is observed [102].

## Appendix H. Overfed Fermentation of *U. cynodontis* ITA Max pH with Mg(OH)_2_

To evaluate the potential of Mg(OH)_2_ as a base in fermentation (Section 3.1.2), a fermentation is performed with Mg(OH)_2_ instead of NaOH (Figure A10). While ITA production is similar to fermentations with NaOH, cell growth stagnates after 69.4 h of the fermentation. In combination with a similar ITA productivity, compared to fermentations with NaOH, the resulting low CDW leads to an overall process yield of 0.48 g_ITA_/g_glucose eq._, without including leftover sugar in the calculations. MgOH_2_ is fed as a suspension, which can result in blocking of tubing and pipes, as is visible between 48 h and 69.4 h. The blocking is removed and pH control is reestablished after 69.4 h. For future process design, where the base of the fermentation and the acid for crystallization are aligned for a maximum process yield, further research into the influence of different cations resulting from base consumption on growth and productivity of *U. cynodontis* ITA Max pH is necessary.

## Appendix I. Discoloration of Crystals after Purification from Fermentation Broth

To evaluate the influence of a complex substrate on crystal colouration, a fermentation with pure glucose is conducted according to Tehrani et al. (2019) [18] with *U. cynodontis* ITA Max pH and a NH_4_Cl concentration of 4 g/L. ITA is then purified according to Section 3.2.3. However, a decolorization step is omitted. Even without this decolorization step, white crystals are obtained. If fermentation broth obtained in Section 3.2.1 is purified in the same way, it is evident, that the use of thick juice for fermentation leads to increased colorization of ITA cystals and necessitates a decolorization step (Figure A11).

## Appendix J. Influence of Temperature on Decolorization Performance

The influence of temperature on decolorization behavior and ITA adsorption is investigated with artificially prepared broth at a starting pH of 3.6 using the XAD-1180 resin as an example.

## Appendix K. Adsorption of ITA on AC-1 and XAD-1180 in Pure Systems

Additionally to the experiments described in Section 3.2.2, ITA adsorption is characterized in artificially prepared systems for AC-1 and XAD-1180 at different pH values to confirm the ITA species primarily adsorbed and to gain information on potential competitive adsorption of pigments and ITA in artificially prepared broth. Figure A13 depicts mostly fully protonated ITA being adsorbed at the hydrophobic decolorization agents XAD-1180 and AC-1.

## Appendix L. Crystallization Profile in Laboratory-Scale

Crystallization in laboratory-scale is performed with a EasyMax 102 Titration Calorimeter (Mettler Toledo, Columbus, USA) and pH is controlled during the cooling crystallization (Figure A14). In total, 300.4 mL 5 M HCl are added per liter of concentrated ITA solution. This corresponds to a chloride concentration of 1.5 mol/L, which is 60% higher than in process simulations. After crystallization, chloride concentration is increased further to 1.75 ± 0.01 mol/L.

## Appendix M. Additional Data for Fermentation Scale-Up

Figure A15 displays a close-up view of pH and CTR between 14 and 17 h of cultivation in the 100 L fermentation. During the first hours of cultivation, the pH fluctuates slightly. In every control step, it is regulated to 6.5 by addition of NaOH. This is coupled to a change in CO_2_ solubility, resulting in an oscillating CTR. The pH is constant and the CTR stabilized after the drop in OTR indicates the beginning of nitrogen limitation.

## Appendix N. Raman Spectroscopy of Sugars

For pure component models (PCM) of glucose, fructose and sucrose, 10 wt% sugar in water is used. For constructing the ITA PCMs, spectra of ITAH_2_ and ITA^2−^ at pH 1.9 and pH 13.0 are used. For ITAH^−^ a binary spectrum with H_2_O was determined using Multivariate Curve Resolution with Alternating Least Squares (MCR-ALS) [103,104,105,106] as it could not be recorded by itself due to being present in mixture with the other two ITA species at any given pH value. The total mixture hard model is calibrated with mixtures of sugar and ITA in water between 1 and 10 wt% and 3 and 8 wt%, respectively. Raman spectra of sucrose, fructose and glucose for PCM can be found in Figure A16–Figure A18. Prominent peaks are identified in the region of 1200-1500 cm^−1^, where mainly Raman bands caused by -CH_2_ are located. A strong band at 1458 cm^−1^ due to the δ_-CH_2__ bending mode, a broader set of merged peaks around 1300–1440 cm^−1^ due to *r*_-CH_2__ rocking and *w*_-CH_2__ wagging modes, and a medium to strong peak around 1264–1268 cm^−1^ due to τ_-CH_2__ twisting vibrations can be found. Further bands are visible between 1000 and 1200 cm^−1^, where δ_C-O-H_ bending vibrations and ν_C-O_ stretching modes occur. Below 800 cm^−1^, mostly δ_C-H_ bending vibrations and ν_C-C_ stretching modes are present [107,108,109,110].

Additionally, for glucose and fructose, bands are present that are distinctly caused by its isomers. α-D-glucose has a C-O stretching mode at 1040 cm^−1^, while β-D-glucose has a band blue-shifted compared to α-D-glucose at 1063 cm^−1^ [109]. While structurally different, these compounds co-exist in approximate constant ratio when dissolved, due to equilibrium in ring opening and closing behavior at a certain temperature and pH. Hence, for glucose a single PCM is constructed that represents the Raman bands of both the α and β form of glucose in given ratios. Similarly, the fructose spectrum is a mixture of bands of β-D-fructopyranose and D-fructofuranose, which are also represented by a single PCM assuming a constant ratio of these isomers in solution [109,111].

The Raman spectra of ITA can be found in Figure A19. To be able to include the sugars in the IHM approach from Echtermeyer et al. (2021) [93], the corresponding model for ITA is extended to a range of 800 to 1800 cm^−1^. Additional prominent bands for ITA found in the region below 1025 cm^−1^ are ν_C-C_ stretching modes and ν_s,(C-O)_ symmetric stretching vibrations related to the carboxylic acid groups [112,113]. Some of these bands shift to higher wavenumbers at higher pH values due to a higher degree of dissociation of the carboxylic acid groups and presence of ions [112].

## Appendix O. Indirect Hard Modelling and Figures of Merit

The mixture hard model created with IHM is set up in PEAXACT and shown in Figure A21. It is comprised of a total of seven main components represented by PCM: water, three ITA species (ITAH_2_, ITAH^−^, ITA^2−^), sucrose, glucose and fructose. Prior to fitting a Raman spectrum, a pretreatment model is first applied which excludes the region 1545– 1565 cm^−1^ attributed to the oxygen gas peak and the spectral range is reduced to 800–1800 cm^−1^. Secondly a linear baseline model is applied, fitted through the lowest points in the spectrum. One auxiliary PCM is included to compensate for a sloped baseline below 1000 cm^−1^, as shown in magenta (Figure A21).

After the mixture hard model is constructed, its goodness of fit is determined to assess the model quality and accuracy. First of all, the model is fitted to a spectrum known to contain the components present in the model as PCMs. Figure A22 shows the model fitted to a mixture of sucrose, glucose and fructose. The residuals show an absolute maximum value of 131, which is more than an order of magnitude smaller than the intensity (5981) of the maximum peak in the spectrum at 1126 cm^−1^ for the alcohol (δ_COH_) bending vibration of the sugars. Additionally, the RMS residual is calculated (Equation (A15)).

(A15) $$RMS=\sqrt{\frac{1}{N}\cdot \sum_{n}^{N}{\left(y_{model,n}-y_{measured,n}\right)}^{2}}$$

*N* is the number of data points in the spectrum and *y* are the Raman intensities at each point *n*, is calculated as 39.8, which is comparably low compared to the peak intensities in the spectrum.

The following figures of merit are also calculated for the mixture hard model to assess further quality and properties of the constructed model. The calibration performance for a component *i* is determined by the root mean squared error of calibration (RMSEC_i_) [g/g] and the root mean squared error of leave-10%-out cross-validation (RMSECV_i_) [g/g], each calculated as shown in Equation (A16).

(A16) $$RMSEC{\left(V\right)}_{i}=\sqrt{\frac{1}{K}\cdot \sum_{k=1}^{K}{\left(w_{predicted,k,i}-w_{true,k,i}\right)}^{2}}$$

*K* is the total number of data points, *k* included in the calibration and *w* the concentration in g/g of the sample measured. The leave-10%-out cross-validation is achieved by repeating the calibration several times and at every iteration 10% of the data is left out of the calibration for calculating the RMSECV, until each datapoint has been left out at least once. Additionally, also the coefficient of determination, R^2^_*i*_ is calculated. Furthermore, the bias of the achieved calibration for each component is assessed using the stability ϕ_*i*_ [-], as depicted in Equation (A17).

(A17) $$\phi_{i}=\frac{RMSECV_{i}-RMSEC_{i}}{RMSEC_{i}}\cdot 100\%$$

The detection limit [g/g] is calculated in Equation A18 by evaluating 10 measurements of a blank sample consisting of deionized water. The weight fraction, $w_{i,blank}$ and standard deviation, $sigma_{i,blank}$ are determined using the mixture model predictions of the blank samples. With a confidence factor, β of 3, a confidence of 99.86% is obtained when a normal distribution of the measurement errors is assumed [114].

(A18) $$LOD_{i}={\bar{w}}_{i,blank}+\beta \cdot \sigma_{i,blank}$$

The resulting PCMs constructed with IHM consist of 24, 19 and 24 component peaks for sucrose, glucose and fructose, respectively, in the range of 800 to 1800 cm^−1^. To match this range, the existing IHM from Echtermeyer et al. (2021) [93] for ITAH_2_, ITAH^−^ and ITA^2−^ is extended from 1025 to 1800 cm^−1^ to 800 to 1800 cm^−1^. From there, ITA PCMs are constructed with 13, 14 and 16 component peaks, respectively, for ITAH_2_, ITAH^−^ and ITA^2−^.

The PCMs for ITA dissociation, sucrose, glucose, fructose and water are combined in a total mixture hard model (Appendix O). Except for water and ITAH^−^, the RMSEC_i_ and RMSECV_*i*_ values are below 1.218 × 10^−3^ g/g, which indicates that the model has a good accuracy. The ITAH^−^ has lower accuracy, as there is a less accurate model fit in the region between pH 3 and 5 (Figure A19) compared to Echtermeyer et al. (2021) [93]. This also affects the water accuracy, as the calibration has an additional closure constraint, stating that the total sum of the weight fractions should add up to 1. As a result, ITAH^−^ accuracy also lowers the H_2_O accuracy. The ϕ_*i*_ is below 3% for all components and thus indicates an unbiased and stable calibration result. Finally, the Limit of Detection (LOD_i_) shows values that are an order of magnitude lower than the RMSECV_i_’s, indicating a precise model. In conclusion, although similar, the overlapping spectral bands of the three sugars and ITA do not hinder an accurate and precise result in a mixture spectral hard model.

**Table A4:** Figures of merit for the mixture hard model.

Component	R^2^_*i*_ [-]	RMSEC_i_ [×10^−3^ g/g]	RMSECV_i_ [×10^−3^ g/g]	LOD_i_ [×10^−3^ g/g]	ϕ_i_ [%]
ITAH_2_	0.997	0.985	1.018	0.118	3
ITAH^−^	0.985	1.481	1.490	0.020	1
ITA^−2^	0.993	1.056	1.070	0.148	1
Sucrose	0.998	1.208	1.218	<0.01	1
Glucose	0.999	0.551	0.558	<0.01	1
Fructose	0.9997	0.392	0.398	<0.01	2
H_2_O	0.986	2.805	2.842	n.d.^1^	1

^1^ n.d. = not defined.

## Appendix P. Raman Spectra Recorded in 100 L Fermentation with Fluorescence

When the Raman probe is inserted directly into the fermenter at the start of fermentation, fluorescence occurs mostly due to biomass and biological activity and due to pigments present in the complex substrate. As the biomass contributes to fluorescence, the shape of the broad fluorescent signal overlaying the Raman bands changes during fermentation. At the end of fermentation, the fluorescence reaches its maximum, as can be seen from the increased Raman intensity, and has shifted towards higher wavenumbers. In addition, noise increases, which is caused by an overall increase in the magnitude of the fluorescence in the recorded spectra by almost a factor of two (Figure A23).
